# A hybrid approach for regionalization of precipitation based on maximal discrete wavelet transform and growing neural gas network clustering

**DOI:** 10.1038/s41598-025-24400-1

**Published:** 2025-11-18

**Authors:** Xu Tao, Ma Ben, He Cao Yin Xuan, Ali Arshaghi

**Affiliations:** 1 College of Mechanical and Electrical Engineering, Wuyi University, Wuyishan, 354300 China; 2 The Key Laboratory for Agricultural Machinery Intelligent Control and Manufacturing of Fujian Education Institutions, Wuyi University, Wuyishan, 354300 China; 3 Department of Electrical Engineering, CT.C., Islamic Azad University, Tehran, Iran

**Keywords:** China, Clustering, MODWT, Precipitation, Pre-processing, Energy science and technology, Engineering

## Abstract

Understanding the spatiotemporal variability of precipitation is critical for effective water resource planning, particularly in regions with diverse climatic zones such as China. This study presents a hybrid methodology combining the Maximal Overlap Discrete Wavelet Transform (MODWT) and the Growing Neural Gas (GNG) clustering algorithm to regionalize precipitation patterns using monthly data from 123 synoptic stations over a 45-year period (1980–2024). MODWT was applied to decompose the precipitation time series into five frequency-based sub-series (W1–W5 and V5), capturing variability across 2- to 32-month cycles. Shannon entropy was calculated for each sub-series, generating a comprehensive feature set that reflects the temporal complexity at each station. These entropy features were subsequently used as input for the GNG algorithm, which identified 12 homogeneous precipitation clusters. The clustering performance was quantitatively assessed using the silhouette coefficient (SC), where the proposed model achieved a maximum SC value of 0.68, indicating strong inter-cluster separation and intra-cluster compactness. In comparison, clustering performed without MODWT-based preprocessing yielded a lower SC value of 0.56, highlighting the effectiveness of the hybrid approach. Spatial analysis revealed that northern and northwestern China exhibited the highest precipitation variability, particularly in the W3 (8-month) and V5 (trend) components, while southern and southeastern regions demonstrated more stable patterns. The results underscore the value of integrating multiscale temporal analysis with neural-based clustering for robust and interpretable regionalization of precipitation. This framework offers substantial potential for informing water resource management, climate adaptation policies, and infrastructure development under future hydroclimatic uncertainty.

## Introduction

 Precipitation is the most important climatic element that exhibits significant spatial and temporal variability. It is a key factor in the hydrological cycle^1^. China’s climate exhibits substantial variability in precipitation patterns across multiple temporal scales. Alterations in the hydrological cycle are likely to significantly influence the spatial and temporal distribution of rainfall^2^.

Understanding the spatiotemporal variability of precipitation is crucial for effective water resource management, particularly in regions susceptible to drought. Recent analyses indicate significant shifts in precipitation patterns across China from 2005 to 2020. Northern China has experienced a marked decrease in annual precipitation, intensifying drought conditions and challenging agricultural sustainability. Conversely, southern regions have witnessed moderate increases in rainfall, leading to heightened flood risks. These divergent trends underscore the complexity of China’s hydroclimatic dynamics and highlight the necessity for region-specific water management strategies to mitigate the impacts of climate variability on food security and water availability^3^.

This case underscores the critical importance of systematic monitoring and strategic management of water resources in regions exhibiting declining precipitation trends, particularly in northern China^4^. The pronounced spatiotemporal variability of precipitation, coupled with recurring drought episodes, presents a substantial challenge for sustainable water governance across the country^5^. This situation is further exacerbated by escalating water demands driven by rapid population growth and economic development, especially in arid and semi-arid regions where agricultural water consumption remains high.

To address these challenges effectively, it is essential to delineate sub-regional climatic zones characterized by distinct precipitation regimes and hydroclimatic responses. Identifying these zones enables the development of region-specific management strategies tailored to the unique conditions of each area. Such a targeted approach is fundamental to fostering more resilient and adaptive water resource planning, ultimately contributing to the long-term sustainability and equitable distribution of China’s water resources^4^. In recent decades, numerous studies have focused on analyzing precipitation patterns across China, aiming to uncover temporal and spatial variability, as well as underlying trends.

These investigations have provided critical insights into regional hydrological behavior, thereby supporting policymakers in designing targeted and adaptive strategies for sustainable water resource management under conditions of increasing water stress. For instance, Jiang et al.^6^ employed spectral clustering techniques to identify synoptic-scale atmospheric patterns associated with extreme precipitation events in the Yangtze–Huai River region.

Their findings revealed four distinct meteorological configurations, each characterized by specific intensities, spatial extents, and geographic locations of heavy rainfall^6^. Similarly, Wu et al.^6^ examined daily precipitation records within the Yangtze River Basin and reported a growing frequency and intensity of extreme rainfall, along with clearly defined spatial trends^7^. In another study, Wang et al.^7^ applied machine learning approaches to detect variations in heavy precipitation across southern China, showing an upward trend in intensity and associating these events with distinct atmospheric circulation patterns^8^.

Collectively, these studies highlight a discernible intensification of extreme precipitation events throughout China, underpinned by well-defined spatiotemporal structures. The wavelet transform is a signal processing approach that transforms data on time-frequency space, making it useful for identifying the predominant frequency of variability and changes over time scales.

Wavelet transform is an effective method for decomposing the localized alteration of a signal, and its efficiency has been demonstrated in clustering-based research studies. Wavelet analysis is a powerful tool for modeling, but its application can be complex and prone to errors. In some cases, improper use of wavelet analysis has led to erroneous outcomes^9^. One common mistake made by some researchers in using wavelet analysis is the decomposition of data into clusters created by wavelet transform. The correct application of wavelet analysis involves isolating and extracting related Characteristics from a given data using wavelet transform^10^.

Despite the extensive body of research dedicated to identifying the spatial and temporal patterns and trends of precipitation in China, the application of the Generalized Growing Neural Gas (GNG) algorithm for regional precipitation clustering has not yet been explored. Most existing studies have predominantly relied on conventional techniques such as Principal Component Analysis (PCA), Hierarchical Clustering Analysis (HCA), or basic wavelet approaches.

However, the integration of advanced hybrid methods—particularly those leveraging machine learning algorithms—for analyzing complex and multi-scale precipitation dynamics remains relatively underutilized. This study introduces a novel analytical framework that incorporates coefficients derived from the Maximal Overlap Discrete Wavelet Transform (MODWT) to classify synoptic stations based on the temporal characteristics of precipitation. The innovation of this research lies in the use of the GNG algorithm for spatial precipitation regionalization, offering enhanced capabilities in detecting nonlinear structures and intricate temporal interdependencies compared to traditional clustering methods. The central objective of the proposed hybrid approach is to improve the accuracy and robustness of spatial clustering by eliminating sub-series that exhibit weak correlation with the original monthly precipitation time series. This is accomplished through the extraction of multi-scale precipitation features from the MODWT output, thereby optimizing the spatial classification process. Consequently, this study addresses a notable gap in the literature and introduces a pioneering methodology for spatial-temporal precipitation modeling in China, with promising implications for water resource management and hydrological risk assessment under changing climate conditions.

## Materials and methods

**Fig. 1:**
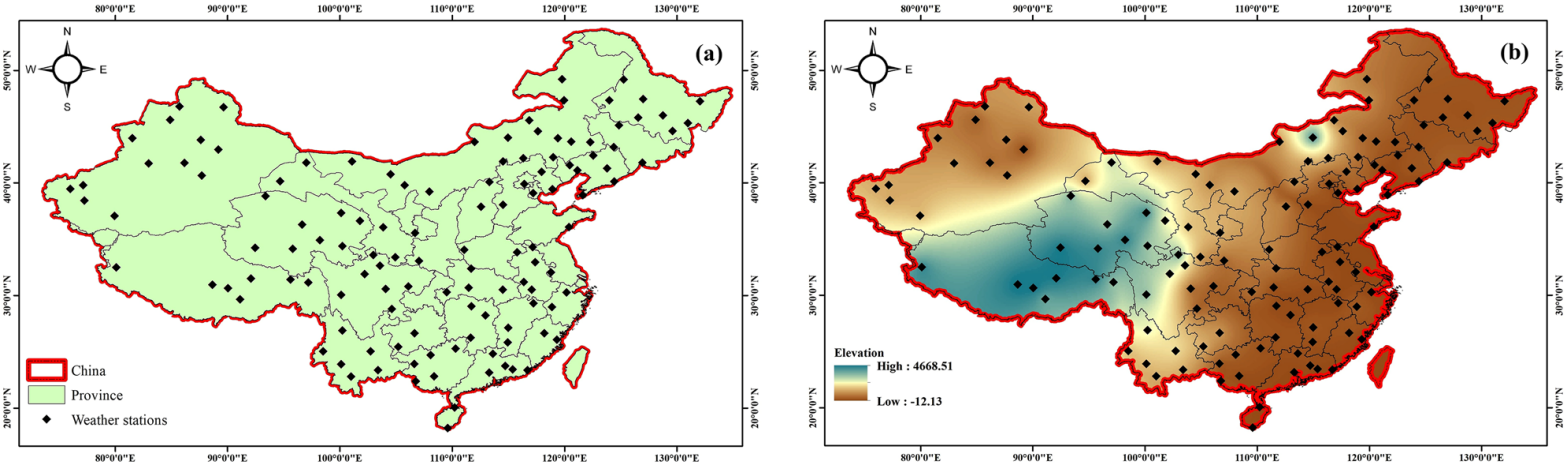
(**a**) Map of China’s location of synoptic stations. (**b**) Map of China’s Digital elevation model.

China, one of the largest countries in East Asia, encompasses a broad spectrum of climatic zones and physiographic conditions due to its vast territorial extent of approximately 9,600,000 square kilometers. This makes it the fourth largest nation globally in terms of land area and a major component of the Asian continental landmass. The country exhibits substantial altitudinal variation, with an average elevation of around 1,840 m above sea level. This elevated topography is primarily attributed to the presence of extensive mountainous and plateau regions, particularly in western China. Topographic elevation varies markedly across the country. The highest point is Mount Everest (8,848 m), located in the Himalayas along the Nepalese border. Other notable mountain ranges include the Tianshan range in the northwest and the Hengduan range in the southwest. Several prominent peaks such as Minya Konka (7,556 m), Muztagh Ata (7,546 m), and Mount Kailash (6,638 m) further contribute to China’s complex geomorphological profile. These elevated terrains exert a substantial influence on the spatial and temporal variability of precipitation patterns across the nation. The combination of mountainous highlands, expansive basins, and lowland plains creates a highly heterogeneous climatic environment, as demonstrated in Fig. 1. To construct reliable hydrological models capable of reflecting such environmental diversity, this study utilized monthly precipitation time series. These datasets were chosen to capture the spatiotemporal heterogeneity inherent in China’s precipitation dynamics. While official reports estimate China’s mean annual precipitation at approximately 645 mm, this research re-evaluated the climatological data for the period 1980–2024 and determined the average to be around 670 mm. Precipitation extremes vary significantly across regions: arid zones in the northwest receive less than 50 mm annually, whereas southeastern coastal areas and the southern flanks of the Himalayas can receive over 2,000 mm.

The present study employed precipitation records sourced from the China Meteorological Administration (CMA), encompassing 123 weather stations distributed across diverse climatic regions, as depicted in Fig. 1. The station selection process was guided by the objective of achieving comprehensive spatial representation, ensuring that each distinct climatic zone was covered by at least one monitoring site. A further criterion for inclusion was the availability of consistent, long-term observational data spanning four decades (1980–2024). High-quality, uninterrupted meteorological datasets are fundamental to ensuring robust statistical assessments in environmental and atmospheric research domains. To evaluate the temporal consistency and homogeneity of the collected precipitation series, the non-parametric Run test was conducted at a 95% confidence level, in accordance with the approach recommended by^11^. This test is frequently used in climatological and hydrological studies to determine whether a sequence of data exhibits random distribution, thereby supporting its suitability for advanced modeling and analytical applications.

### Maximal overlap discrete wavelet transforms (MODWT)

In 1992, Daubechies introduced the Wavelet Transform (WT) as a method for decomposing a signal into various time scales, using a set of mother wavelets as basic functions^12^. This approach can reveal hidden information from the original signal, such as discontinuities, inflection points, and trends that may not be evident in the raw signal. DWT and CWT are two types of wavelet transforms^13^. However, since precipitation time series are recorded at discrete time intervals, DWT is preferred for hydrological signal decomposition^14^. The MODWT is a mathematical method that converts a signal into multiple levels of wavelet and scaling coefficients. MODWT provides several advantages over DWT, as discussed in Cornish et al.^15^. For example, MODWT can be used with signals of arbitrary lengths, while DWT is limited to signals with lengths that are integer multiples for a power of two. The MODWT concept involves breaking down a signal into overlapping segments and applying a wavelet transform to each segment individually. The wavelet coefficients obtained for each segment are then combined to obtain the final wavelet coefficients for the entire signal. The MODWT is commonly used for time series decomposition due to its ability to consider boundary conditions (BCs) when decomposing data, which helps avoid errors in developing of forecasting models^16^. Previous have shown the impact of BCs on time series decomposition and the potential for incorrect predictions if BCs are not appropriately addressed^17^. The MODWT is defined based on the DWT, where (hj, k) represents the DWT filter and ($\:\widehat{g}$_j, k_) represents the scale filter, with k = 1…, representing the filter length (L), and j levels of decomposition. The MODWT wavelet filter ($\:\widehat{h}$_j, k_) and the MODWT scale filter ($\:\widehat{g}$_j, k_) are defined as $\:\widehat{h}$j, k = hj, k / 2j/2 and $\:\widehat{g}$j, k = gj, k / 2j/2, respectively. The wavelet coefficients of MODWT at level j are defined as the deviation of the time series (Xt), and the MODWT filters are obtained using Eqs. (1) and (2).

1 $$\:{\stackrel{\sim}{W}}_{j,t}={\sum\:}_{k=0}^{{K}_{j}-1}{\stackrel{\sim}{h}}_{j,k}{X}_{t-kmodN}$$

2 $$\:{\stackrel{\sim}{V}}_{j,t}={\sum\:}_{k=0}^{{K}_{j}-1}{\stackrel{\sim}{g}}_{j,k}{X}_{t-kmodN}$$

In the equations, $\:{\stackrel{\sim}{W}}_{j,t}\:$represents the wavelet coefficient, Ṽj, t represents the scale coefficient, modN represents the modulo operation when treating the historical series as periodic with periods equal to N, and K_j_ is obtained using Eq. (3).

3 $$\:{K}_{j}=\left({2}^{i}-1\right)\left(K-1\right)+1$$

Amount of Kj represents the number of wavelet coefficients and scales influenced by boundary conditions (BC) for decomposition level J and wavelet filter length level K. This equation enables the acquisition of wavelet and scale coefficients that have been adjusted for limits, thus avoiding the introduction of additional uncertainty to these coefficients caused by future data issues^17^. The MODWT employs a high-pass filter ($\:\stackrel{\sim}{h}$) to compute its wavelet coefficients, and it utilizes an iterative method to construct the time series (Xt), which can be reconstructed using Eq. (4).

4 $$X_{t} = \tilde{W}_{{j,t}} + \tilde{V}_{{j,t}}$$

**Fig. 2:**
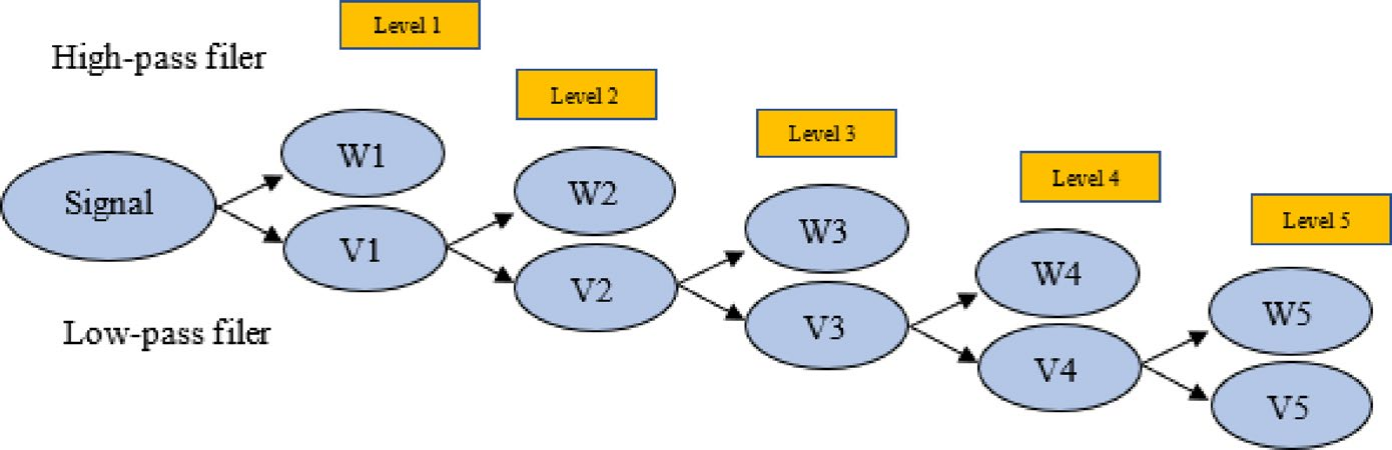
Flowchart for five-level MODWT used for monthly precipitation data.

Figure 2 depicts a flowchart of a five-level precipitation data decomposition by MODWT. The MODWT-based Multiresolution Analysis decomposes an original precipitation signal into scaling (V5) and coefficients (W1, W2, W3, W4, and W5), as illustrated in the figure. This procedure involves dividing the precipitation signal into overlapping segments and applying wavelet transforms to each segment individually. The scaling coefficients show the low-frequency components of the precipitation signal, while the wavelet coefficients represent the high-frequency components^18^.

### Daubechies wavelets

The Daubechies function is a commonly used mother wavelet in the field of wavelets for signal processing and analysis. The wavelets in the Daubechies family are denoted as dbN, where N is the order and db refers to the wavelet name. The Daubechies wavelets are both orthogonal and biorthogonal and have associated minimum-phase scaling filters, making them suitable for various signal processing applications^19^. However, they do not have a straightforward analytical expression, except for the db1 form, which is equivalent to the Haar wavelet^20^. In this study, the db4 mother wavelet was used to decompose the precipitation time series, since the length of the time series is 492. Due to the selection of appropriate boundary extension and decomposition level, taking into account, it was verified that the db4 mother wavelet produced the most accurate reconstructed time series for all of the synoptic stations than other wavelet selections.

The wavelet selection parameters were also evaluated based on previous studies that conducted opening similar precipitation data and taking into consideration to do similar methods of choosing a wavelet and decomposition level that would effectively decompose trends and cycles. Probably most notably, a sensitivity analysis was conducted to ensure the decomposition level did capture the main temporal changes with no overfitting or significant data loss. Overall, this information provided verification that previous studies could and does create an effective analysis of the study area.

### Boundary conditions for MODWT-based analysis

There are two methods for addressing boundary effects in wavelet analysis: modifying the wavelets or modifying the data. Cohen et al.^21^ proposed the “wavelet on the interval” method, which modifies the wavelets. In this study, the method used involves modifying the data. In order to apply the MODWT algorithm, an infinite time series is required. However, real-world data, such as precipitation measurements, are typically recorded at discrete time intervals and within a limited time frame. To apply wavelet analysis, the focus is on the right end of the series because the left end is affected by the boundary condition. To address this, there are two standard methods that can be applied. One approach is to extend the time series periodically. This involves defining a new time series by repeating the observed values at the beginning and end of the precipitation time series. For example, if the original time series is P1, P2, P3, ., PN, the periodic extension would be P0, P1, P2, P3, ., PN, PN + 1, PN + 2, ., 2 N-P1, 2 N-P2, ., 2 N-PN. The periodic extension assumes that the time series is periodic, which may not be appropriate for all applications. The second approach is to mirror-extend the time series, which involves reflecting the time series about the first and last observed values. For example, if the original time series is P1, P2, P3, ., PN, the mirror extension would be P3, P2, P1, P1, P2, P3, ., PN, PN-1, PN-2, ., P3, P2, P1. The mirror extension assumes that the time series is symmetric about the observed values, which may be more appropriate for some applications. Both methods can be used to extend the time series and address the boundary condition in wavelet analysis, but the choice of method depends on the specific application and the characteristics of the time series. The sub-series obtained through MODWT are used as inputs for the GNG model. However, this approach may not consider the values affected by the boundary conditions on the left end of the series, which can affect the model accuracy^18^.

### Growing neural gas network (GNG)

The GNG algorithm is an unsupervised clustering method based on artificial neural networks^22^. The GNG algorithm is distinct from classical clustering algorithms in that it constructs a network structure that is inherently compatible with the topology of large datasets via topology learning^23^. The algorithm continuously introduces new nodes (neurons) to an initial small network, resulting in a growing structure. In the GNG network, neurons compete with each other to identify the closest similarity to the input dataset^24^.

The GNG algorithm can be summarized in the following steps:

1. Initialize a small network with two nodes (w1 and w2).
2. Select an input pattern from the dataset randomly.
3. Find the two closest nodes to the input pattern in the network (s1 and s2).
4. Increase the age of all edges connected to s1. This measures the strength of links between neurons.
5. Increase the accumulated error in s1. This identifies areas where neurons do not sufficiently match input vectors.
6. Move s1 and its direct topological neighbors towards x by learning rates εb and εn, respectively, adapting the network to the input space (Eqs. 5 and 6):
  5 $$\:{\varDelta\:w}_{s1}={\epsilon\:}_{b}(x-{w}_{s1})$$
  6 $$\:{\varDelta\:w}_{n}={\epsilon\:}_{n}(x-{w}_{n})$$
7. If s1 and s2 are connected by an edge, set the edge age to zero. If not, create an edge. This inserts edges between the two closest neurons, constructing network topology.
8. Remove edges with an age over amax. Remove isolated points. This eliminates unnecessary connections between neurons.
9. If the number of input vectors is an integer multiple of λ, insert a new neuron halfway between the neurons q and f with the largest error variables. Decrease the error variables of q and f. This inserts new neurons in areas of the input space indicated by the accumulated error.
10. Decrease all error variables by a fraction β.
11. Repeat steps 2 through 10 until a stopping criterion is met, such as a maximum number of iterations or a minimum error threshold.

The GNG algorithm has been successfully applied in various domains, including data clustering, visualization, and anomaly detection^25^. Overall, the GNG algorithm is an unsupervised neural network-based clustering algorithm that develops a network topology compatible with learning the structure of large datasets.

### Kmeans clustering method

The K-means method is the simplest and perhaps most broadly used and well-known unsupervised clustering algorithms available. K-means can be very effective for clustering numeric values, such as temperature and precipitation^26^. K-means was first published in MacQueen^27^, but it is acknowledged to have been first described in original form by Steinhaus^28^. The primary goal of the K-means algorithm is to minimize the within-cluster sum of square errors, that is expressed the following function:

7 $$\:J=\sum\:_{i=1}^{k}\sum\:_{i=1}^{n}{‖{X}_{i}^{j}-{C}_{l}‖}^{2}$$

Where Xji refers to temperature or precipitation data points, Cl means cluster center, and n is the number of data points. Steps of the K-Means Algorithm^29^:

(1) Randomly select K initial cluster centers, (2) Assign each data point to the nearest center based on the Euclidean distance from the data point to the center, (3) Recalculate the cluster centers as the mean of the data points assigned to each cluster, (4) Return to steps 2 and 3 until cluster centers no longer change Having many clusters can lead to a more complex model and may require a larger dataset to reliably estimate parameters.

The standard K-means algorithm is designed for numerical data and relies on the Euclidean distance metric to calculate distances; however, extended formulations of the K-means algorithm have been created to use other distance measures (e.g. Manhattan or binary distance) when clustering non-numeric or categorical data.

### Self-organizing map (SOM)

Kohonen introduced the Self-Organizing Map (SOM) in 1981, a type of unsupervised Teuvo neural network that mimics the shape of retinal neurons. It enables high-dimensional data to be represented in two dimensions for human interpretation and understanding, allowing us to visualize relationships and similarities easily. SOM is a nonlinear clustering technique that utilizes proximity and representational (interpretable) data and it is not like the K-means clustering method^30^. SOM keeps the spatial and metric relationship, projecting complex data into lower dimensions while using similarity relationship for representations or effective cluster information for clustering^31^.

The training is comprised of two iterative processes: Best Matching Unit (BMU) Selection, and Weight updates with neighborhood adaptation. SOM Steps for Trainin^32^:

*Step 1: Initialization*.

- Specify how many neurons are in the input layer (n) and the output layer (m).
- Initialize a network with a grid topology in 2 dimensions.
- Initialize the connection weights from the input layer to the output layer (the weights should be chosen randomly from [0:1]).

*Step 2: Distance Calculation*.

Compute the Euclidean distance between the input vector Xk​ and each neuron’s weight vector Wj​:

8 $$\:{d}_{jk}=‖{X}_{k}-{W}_{j}‖=\sqrt{\sum\:_{i=1}^{n}{X}_{ki}-{W}_{ji}}$$

Identify the winning neuron (BMU) with the smallest distance:

9 $$\:‖{X}_{k}-{W}_{j}‖=min\left\{{d}_{jk}\right\}$$

*Step 3: Weight Update*.

Update the weights of the winning neuron and its neighbors using:

10 $$\:{W}_{j}\left(t+1\right)={W}_{j}\left(t\right)+\mathrm{ղ}\left(t\right){h}_{jc}\left(t\right)\left[{X}_{k}\left(t\right)-{W}_{j}\left(t\right)\right]$$

Where η(t): learning rate, and h_jc_(t): neighborhood function, typically Gaussian:

11 $$\:{h}_{jc}\left(t\right)=exp\left(\frac{‖{r}_{c}-{r}_{j}‖}{2{\sigma\:}^{2}\left(t\right)}\right)$$

r_c_​: position of BMU, r_j_​: position of neuron j, and σ(t): neighborhood radius.

*Step 4: Learning Rate and Neighborhood Radius Decay*

12 $$\:\mathrm{ղ}\left(t\right)=\mathrm{ղ}(1-\raisebox{1ex}{$t$}\!\left/\:\!\raisebox{-1ex}{$T$}\right.)$$

13 $$\:\left\{\begin{array}{c}\sigma\:\left(t\right)=\sigma\:.exp(-\raisebox{1ex}{$t$}\!\left/\:\!\raisebox{-1ex}{$\tau\:$}\right.)\\\:{h}_{jc}=exp(-\frac{{d}_{ij}^{2}}{2\sigma\:{\left(t\right)}^{2}})\end{array}\right.$$

τ: time constant.

As t increases, the neighborhood radius σ(t) shrinks to zero, refining the map resolution.

The training goes on until a maximum number of iterations is achieved, yielding the resulting two-dimensional SOM map, in which the similar patterns are nearer to each other and the dissimilar patterns are farther apart.

### Evaluation criteria

The silhouette coefficient was employed as a means of evaluating the caliber of the spatial clustering output produced by the GNG algorithm. It served as a measure of cluster validity^33^. The SCi (silhouette coefficient index) measures the degree of similarity between synoptic stations in a cluster, with a greater value indicating more precise differentiation among clustering outcomes. To compute the SCi, Eq. 14^34^.

14 $$\:SCi=\frac{b\left(i\right)-a\left(i\right)}{max\left\{a\left(i\right),b\left(i\right)\right\}}$$

where a(i) is the average dissimilarity between a synoptic station and all other synoptic stations in the same cluster, and b(i) is the minimum average dissimilarity between the synoptic stations and all other clusters. The SCi ranges from − 1 to 1, with a value of 1 indicating that the synoptic station is well matched to its own cluster and poorly matched to neighboring clusters.

A value of 0 indicates that the synoptic station is equally similar to two different clusters, while a negative value indicates that the synoptic stations is more similar to neighboring clusters than to its own cluster. The silhouette coefficient provides a quantitative measure of the quality of the clustering result and can be used to compare the performance of different clustering algorithms or parameter settings.

Davies–Bouldin Index (DBI) is an internal cluster quality assessment metric that is widely used in hydrology, because it can determine the best possible well-separated and compact clusters^35^. The Davies–Bouldin Index (DBI) is a function of the ratio of within-cluster scatter to separation between clusters and is given by Eq. (15):

15 $$\:DB=\frac{1}{\mathrm{k}}{\sum\:}_{i=1}^{k}{max}_{j=1,\dots\:,k},\:i\ne\:j\left\{\frac{diam\left({C}_{i}\right)+diam\left({C}_{j}\right)}{‖{C}_{i}-{C}_{j}‖}\right\}$$

Here, the cluster diameter is calculated using Eq. (16):

16 $$\:diam\left({C}_{i}\right)={\left(\frac{1}{{n}_{i}}\sum\:_{x\in\:{c}_{i}}{‖x-{z}_{i}‖}^{2}\right)}^{\frac{1}{2}}$$

In Eq. (9), n_i_ is the number of points in cluster C_i_, and z_i_ is the centroid of cluster C_i_. The objective is to find clusters that have the smallest distance within the cluster and the largest distances from all other clusters. Thus, the best clustering will produce the smallest Davies–Bouldin Index score.

The Calinski–Harabasz (CH) Index is another common coefficient for evaluating clustering algorithms’ performance. Calinski and Harabasz proposed this index in 1974. The Calinski-Harabasz Index, similar to the Silhouette Index, is based on two elements, with one representing the degree of cohesion of data points assigned to a cluster and the other representing the degree of separation between clusters.

For this reason, the Calinski-Harabasz Index is an evaluation coefficient based on the ratio of between-cluster dispersion to within-cluster dispersion based on Eq. (17)^36^:

17 $$\:CH\left(\mathrm{K}\right)=\frac{B\left(K\right)(N-K)}{W\left(K\right)(N-K)}$$

In the equation above, K denotes the number of clusters and NNN denotes the total number of samples. B(K) refers to the between-cluster dispersion matrix, and W(K) is the within-cluster dispersion matrix. Their calculations can be accomplished using Eqs. (18) and (19), respectively.

18 $$\:B\left(K\right)=\left(\sum\:_{k=1}^{K}{a}_{k}{‖{\stackrel{-}{X}}_{k}-\stackrel{-}{X}‖}^{2}\right)$$

19 $$\:W\left(K\right)=\left(\sum\:_{k=1}^{K}{\sum\:}_{c\left(j\right)=k}{‖{\stackrel{-}{X}}_{k}-\stackrel{-}{X}‖}^{2}\right)$$

### Suggested regionalization methodology

Regionalization of synoptic stations in this study was conducted in three stages: pre-processing, spatial clustering, and model verification. The input data structure significantly impacts clustering outcomes, as precipitation time series often exhibit disorderliness, non-stationarity, and dynamic features varying over time^18^. Therefore, a methodology capturing multiscale data features is needed to determine homogeneous clustering areas^37^. This study proposes a spatio-temporal methodology capturing dynamic features in the data, such as MODWT-transformed precipitation time series. Precipitation time series contain useful information, but noise can mislead spatial or temporal analysis. Pre- or post-processing, such as using MODWT to decompose time series into scales, is recommended^16^. An effective regionalization methodology would use temporal pre-processing and spatial clustering, considering multiscale precipitation data representations simultaneously. Here, precipitation time series were decomposed using MODWT to capture multiscale features, used as GNG spatial clustering algorithm inputs. In MODWT, the Daubechies 4 mother wavelet was used. Decomposition level and boundary treatments were optimized for accurate, robust regionalization. MODWT generates V5 and Wi coefficients, not all highly correlated with precipitation. The clustering input layer was optimized by selecting V5 and Wi coefficient combinations capturing various temporal periods. These were GNG algorithm inputs to spatially recognize homogeneous precipitation regions. Model validation evaluated clustering accuracy and effectiveness. Overall, the proposed methodology systematically regionally synoptic stations, incorporating temporal pre-processing and spatial clustering, applicable to similar datasets. MODWT captured useful precipitation time series features at multiple scales, used in optimized GNG clustering inputs to accurately determine homogeneous precipitation areas. Comprehensive spatio-temporal analysis generated clustered regions reflecting data characteristics. Systematic validation assessed how well the clustered precipitation regions matched the dataset features.

### Wavelet coherence

Wavelet coherence is a method of examining the relationship between two signals and is particularly effective in the analysis of non-stationary time series, such as precipitation. For two finite-energy signals, x(t) and y(t), classical cross-correlation defines the interaction of these signals^38^. Using wavelet transform to analyze precipitation or teleconnection signals, their spectral dependence is articulated as:

20 $$\:{\uprho\:}\left(f\right)=\frac{{S}_{xy}\left(f\right)}{\sqrt{\left|{S}_{xx}\left(f\right){S}_{yy}\left(f\right)\right|}}$$

where Sxy(f) is the cross-spectral density between the precipitation and the teleconnection signal. As precipitation series tend to be non-stationary in nature, the use of time-frequency representations is suggested. In order to overcome the issues with using classical coherence on a non-stationary data set (which can yield a value of 1, even if there is no real correlation), wavelet coherence is adopted^39^. Torrence and Webster (1999) provided a way to smooth the wavelet spectrum, given by:

21 $$\:{\mathrm{S}\mathrm{w}}_{\mathrm{x}\mathrm{x}}\left(\mathrm{a},{\uptau\:}\right)={\int\:}_{\mathrm{t}-{\updelta\:}/2}^{\mathrm{t}+{\updelta\:}/2}{\mathrm{W}}_{\mathrm{x}\mathrm{x}}^{\mathrm{*}}\left(\mathrm{a},{\uptau\:}\right){\mathrm{W}}_{\mathrm{x}\mathrm{x}}\left(\mathrm{a},{\uptau\:}\right)\:\mathrm{d}\mathrm{a}\mathrm{d}{\uptau\:}$$

22 $$\:{\mathrm{S}\mathrm{w}}_{\mathrm{x}\mathrm{y}}\left(\mathrm{a},{\uptau\:}\right)={\int\:}_{\mathrm{t}-{\updelta\:}/2}^{\mathrm{t}+{\updelta\:}/2}{\mathrm{W}}_{\mathrm{x}\mathrm{x}}^{\mathrm{*}}\left(\mathrm{a},{\uptau\:}\right){\mathrm{W}}_{\mathrm{y}\mathrm{y}}\left(\mathrm{a},{\uptau\:}\right)\:\mathrm{d}\mathrm{a}\mathrm{d}{\uptau\:}$$

Here δ is the scalar which defines the size of the 2D smoothing window. Wavelet coherence is computed similarly as:

23 $$\:\mathrm{W}\mathrm{C}\left(\mathrm{a},{\uptau\:}\right)=\frac{\left|{\mathrm{S}\mathrm{W}}_{\mathrm{X}\mathrm{Y}}(\mathrm{a},{\uptau\:})\right|}{\sqrt{\left[\left|{\mathrm{W}}_{\mathrm{X}\mathrm{X}}(\mathrm{a},{\uptau\:})\right|\left|{\mathrm{W}}_{\mathrm{Y}\mathrm{Y}}(\mathrm{a},{\uptau\:})\right|\right]}}$$

The value of WC(a,τ) varies between 0 and 1, allowing for the discovery of periodicities and time-localized relationships between precipitation and teleconnection signals^38^.

In this study, wavelet coherence analysis was employed to examine the correlation between precipitation at 12 selected stations—each representing a distinct cluster—and large-scale climate patterns, including the Pacific Decadal Oscillation (PDO), Pacific–North American pattern (PNA), El Niño–Southern Oscillation (ENSO), and North Atlantic Oscillation (NAO).

## Results and discussion

### Results of spatial clustering monthly precipitation data

In this study, we employed the GNG clustering algorithm to identify homogenous clusters and determine the optimal number of clusters for 123 synoptic stations in China. The historical monthly precipitation data spanning 45 years (1980–2024) for each synoptic station was used to train the GNG algorithm and facilitate the clustering process.

The GNG algorithm was chosen for its ability to capture the topological structure of the plan and provide an overview of the resulting clusters. To use the GNG algorithm for regionalizing precipitation data, historical monthly precipitation data from different locations are first collected. The GNG algorithm is then employed to cluster the data based on the similarities in precipitation patterns.

The algorithm works by creating a network of nodes that represent different clusters, and then adjusting the network structure based on the input data. The algorithm uses a competitive learning rule to update the network by adding new nodes and adjusting the weights of existing nodes.

**Fig. 3:**
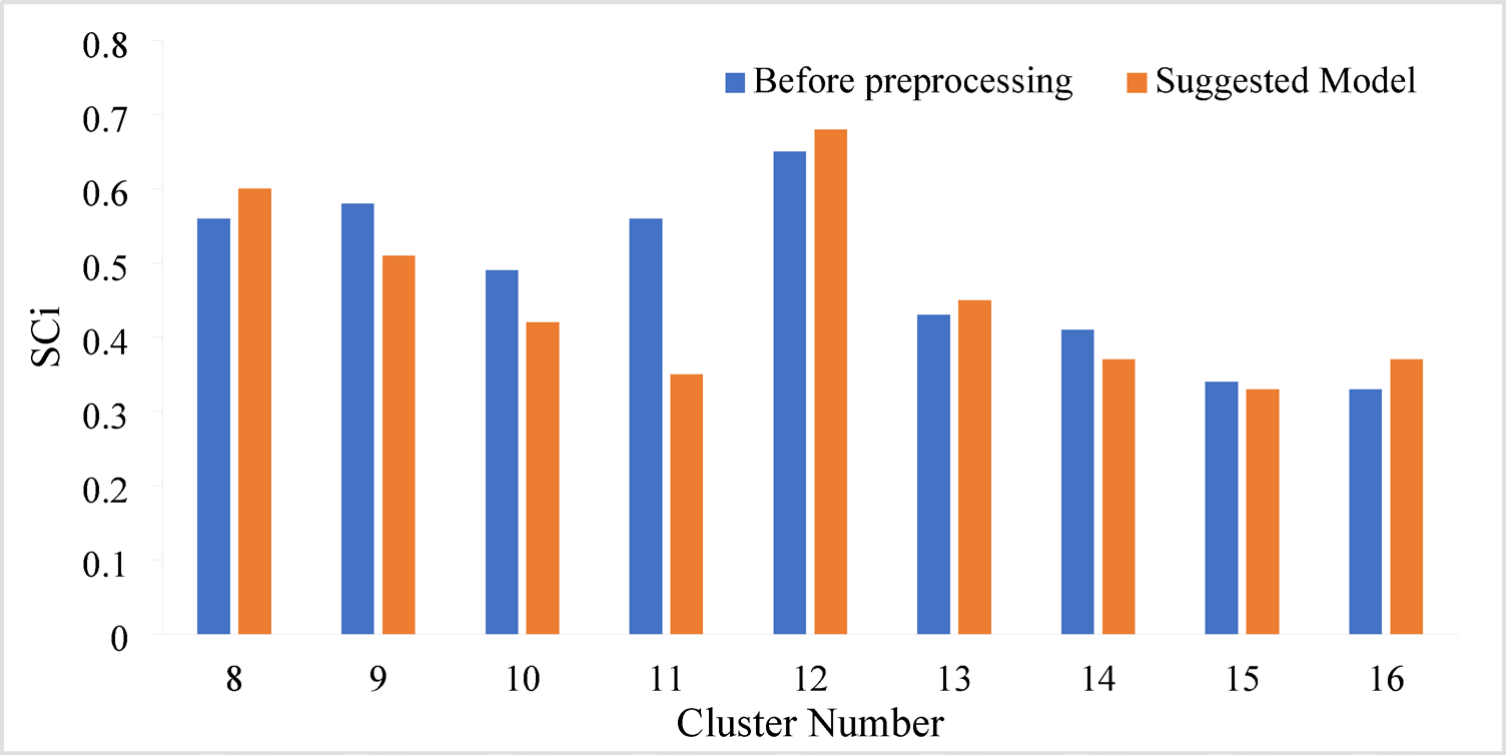
Method analysis for optimal cluster number based on multiple validity indices.

Once the GNG algorithm has been applied to the precipitation data, the resulting clusters can be analyzed to identify homogenous areas. To validate the GNG technique results, SCi validity indexes were employed to determine the optimal number of clusters. The historical dataset was used to cluster the synoptic stations using the GNG technique, and the results are presented in Table 1; Fig. 3.

According to the Table 1, The proposed model via the GNG method provided the most superior clustering performance for 12 clusters. The Silhouette index rose from 0.65 before preprocessing to 0.68 (Fig. 3); the Davies-Bouldin index dropped from 1.10 before preprocessing to 0.91 and the Calinski-Harabasz index increased from 17.75 to 18.98, which reflects a considerable degree of separation between clusters and degree of cohesion within clusters.

The Kmeans method also improved substantially via the proposed model for 12 clusters; its Silhouette coefficient increased to 0.61, the Davies-Bouldin index improved from 1.15 to 1.04 and the Calinski-Harabasz index improved from 17.82 to 18.01. The self-organizing maps (SOM) method underperformed compared to the other two methods, but it did produce positive changes via the proposed model for 12 clusters; its Silhouette coefficient was 0.52, the Davies-Bouldin index was 1.05 and the Calinski-Harabasz index was 17.92, with marginal improvements over its earlier measures.

The proposed model improved the clustering indices for all three methods, while the best number of clusters for the sample data studied was 12 as noted by the highest clustering quality in the metric evaluations. Hence, the data was clustered into 12 group with an optimal value of SCi = 0.68 for historical classification of synoptic station data. Moreover, the study aimed to assess the effectiveness of the proposed MODWT-based approach for precipitation regionalization and identify any potential enhancements.

The proposed model for precipitation regionalization involves using MODWT to preprocess monthly precipitation time series data and extract coefficients. This is followed by using the coefficients to determine the input coefficients for a GNG algorithm, which clusters regions based on their precipitation patterns. The model consists of three stages: extracting dynamic properties of the data using MODWT, determining the input dataset structure using various combinations of V4 and Wi coefficients, and verifying the methodology’s results. The coefficients obtained from the db4 mother wavelet were specifically chosen for use in this process.

**Table 1:** Results of clustering via historical-based clustering methods and suggested model in terms of evaluation metrics.

Cluster method	Indices	Cluster number	8	9	10	11	12	13	14	15	16
GNG	SCi	Before preprocessing	0.56	0.58	0.49	0.56	0.65	0.43	0.41	0.34	0.33
		Suggested model	0.6	0.51	0.42	0.35	0.68	0.45	0.37	0.33	0.37
	DBi	Before preprocessing	1.59	1.57	1.22	1.15	1.1	1.17	1.05	1.03	1.06
		Suggested model	1.52	1.44	1.1	1	0.91	1.12	0.98	0.95	0.99
	CH	Before preprocessing	17.4	14.25	16.21	13.19	17.75	15.35	15.99	11.67	12.02
		Suggested model	18.79	15.87	16.49	15.49	18.98	16.07	18.41	13.26	13.22
Kmeans	SCi	Before preprocessing	0.3	0.34	0.36	0.35	0.52	0.31	0.32	0.36	0.2
		Suggested model	0.4	0.48	0.49	0.59	0.61	0.41	0.35	0.33	0.23
	DBi	Before preprocessing	1.59	1.53	1.54	1.28	1.15	1.36	1.52	1.36	1.42
		Suggested model	1.56	1.49	1.34	1.16	1.04	1.27	1.42	1.1	1.18
	CH	Before preprocessing	15.86	12.38	12.21	13.78	17.82	18.15	16.06	16.39	16.09
		Suggested model	15.95	13.21	13.5	13.98	18.01	18.2	17.06	17.36	17.21
SOM	SCi	Before preprocessing	0.45	0.34	0.32	0.35	0.58	0.4	0.25	0.21	0.36
		Suggested model	0.56	0.35	0.33	0.37	0.52	0.42	0.33	0.28	0.39
	DBi	Before preprocessing	1.62	1.51	1.35	1.26	1.11	1.25	1.39	1.18	1.19
		Suggested model	1.52	1.46	1.3	1.12	1.05	1.23	1.38	1.08	1.1
	CH	Before preprocessing	15.87	12.39	12.22	13.79	17.75	15.49	16.09	14.84	15.9
		Suggested model	16.02	13.5	13.68	13.85	17.92	16.01	16.34	15.05	16.2

### Decomposition of precipitation time series using MODWT

**Fig. 4:**
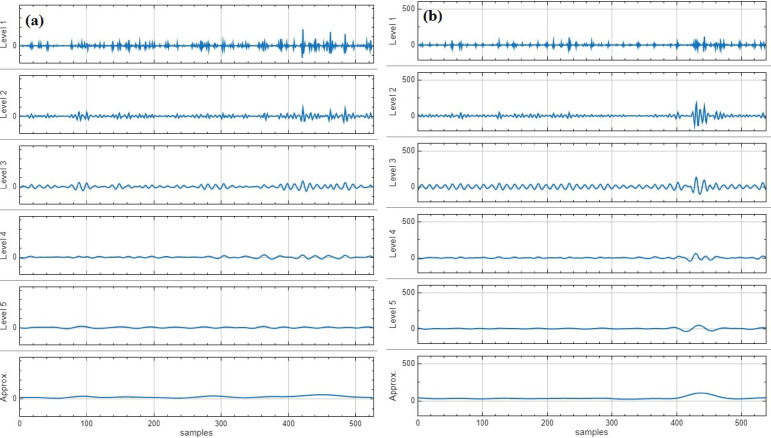
Subseries derived from the decomposition of precipitation data in (**a**) Hotan, and (**b**) Huade weather station using db (4,5).

As shown in Fig. 4, this decomposition corresponds to a time frame of 12 months. The resulting coefficients included one V5 component from the last level of decomposition, and four W coefficients representing each level of decomposition. The five lower resolution levels captured by the coefficients represent cycles of two, four, eight, sixteen, and 32 months, with W1 corresponding to the shortest cycle and W5 to the longest. The V5 coefficient is the approximation component at the fifth level of decomposition in a wavelet transform. Figure 3 demonstrates an example for the application of MODWT to the monthly precipitation data in the Hotan, and Huade weather station. The db4 wavelet and “reflection” boundary treatment for the wavelet decomposition of the precipitation time series implies that the lower wavelet levels capture the high-frequency, rapidly changing components of the dataset, while the higher levels (including V5) capture the low-frequency, gradually changing components of the dataset, such as trend. This is because the db4 wavelet is a high-pass filter that is more sensitive to capturing the high-frequency data details, while the “reflection” boundary treatment allows for better preservation of the trend component of the data. The equation Lj = (2j-1) x (L-1) + 1 was used to remove the first 20 data points from the time series, which were not considered in the modeling process.

### Results of the monthly precipitation regionalization process conducted with hybrid model

**Fig. 5:**
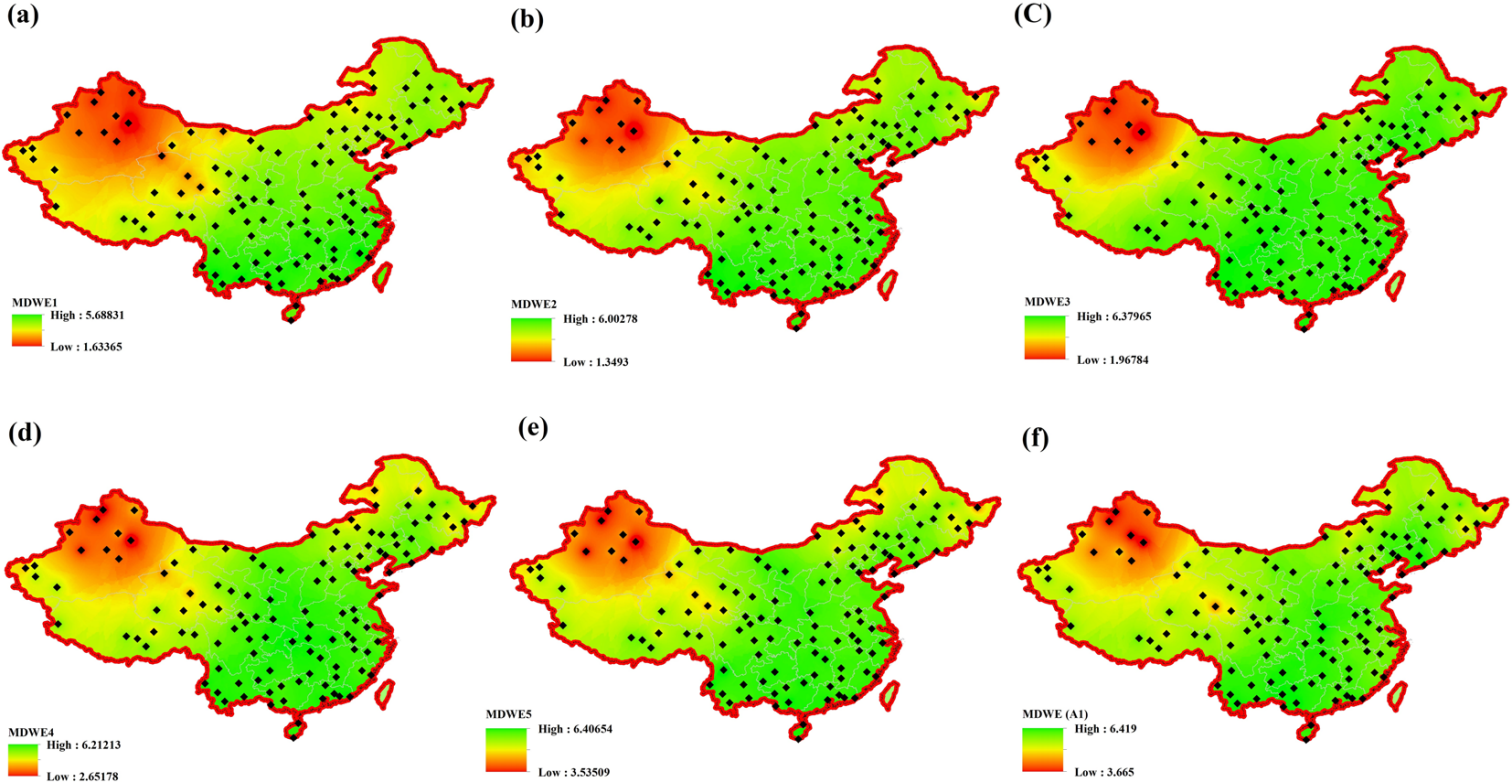
Spatial distribution of monthly wavelet entropy (MDWE) across China based on MODWT-decomposed precipitation sub-series at different time scales.

The process of regionalizing precipitation data involved calculating entropy for the sub-series of precipitation obtained from MODWT-Entropy based coefficients. Figure 5 shows the spatial distribution of entropy changes for five sub-series obtained from MODWT, including partial and approximate sub-series at the monthly scale (MWE). The results indicate that the highest MWE values were calculated for the D3 (eight months) and A5 (trend) sub-series, while the lowest values were obtained for the 1D (two months) sub-series. As observed in Fig. 5, the MWE changes exhibit greater variability at different scales in the north and northwest regions of China. It is noteworthy that these areas are typically characterized by rainy, cold, and occasionally semi-arid conditions. Conversely, the eastern and southern parts of the country, which are primarily dry, exhibit smoother changes. Therefore, it can be concluded that precipitation variability is greater in the north and northwest regions in China.

Figure 5 displays the spatial distribution of Monthly Wavelet Entropy (MWE) across synoptic stations in China, derived from precipitation sub-series obtained using the Maximal Overlap Discrete Wavelet Transform (MODWT). Each subfigure (a–f) represents a different wavelet component associated with distinct temporal scales, ranging from short-term oscillations to long-term trends. Shannon entropy was applied to each sub-series at each station to evaluate the degree of variability or complexity in precipitation patterns over time.

In subfigure (a), corresponding to the W1 component (approximately 2-month cycles), the highest entropy values appear in northern and northeastern China, indicating significant short-term precipitation variability in these regions. In contrast, lower entropy values are observed in the western areas, suggesting more stable short-term rainfall patterns. Subfigure (b), which shows entropy values for the W2 component (around 4-month cycles), exhibits a similar spatial pattern.

However, the high-entropy region extends further across the eastern belt, implying increased variability in intra-seasonal rainfall in these areas. Subfigure (c), related to the W3 component (roughly 8-month cycles), shows the highest entropy values across all wavelet levels. This suggests that annual-scale precipitation fluctuations are particularly prominent in northeastern China, potentially due to regional climate dynamics or seasonal transitions.

In subfigure (d), which represents the W4 component (approximately 16-month cycles), the zones of high entropy continue to dominate the north and northeast, though with slightly reduced intensity compared to W3. This pattern reflects persistent variability on longer temporal scales, possibly driven by multi-year climate phenomena. Subfigure (e) presents entropy values for the W5 component (about 32-month cycles), where spatial differences in entropy become more subtle. Nonetheless, northern and central regions still demonstrate relatively high values, indicating sustained variability in long-term precipitation trends. Lastly, subfigure (f) illustrates the entropy of the A1 component, representing the smoothed approximation or long-term trend of the precipitation time series. This component reflects slow, low-frequency changes. The findings suggest that the western regions exhibit more complex long-term precipitation behavior, while the eastern regions display a more regular trend over time.

Overall, the spatial analysis reveals a consistent pattern of elevated entropy—indicative of greater variability—in northern and northwestern China across multiple temporal scales. These regional disparities are likely influenced by varying climatic conditions, with the north experiencing colder, wetter environments compared to the drier, more stable climates of the south.

**Fig. 6:**
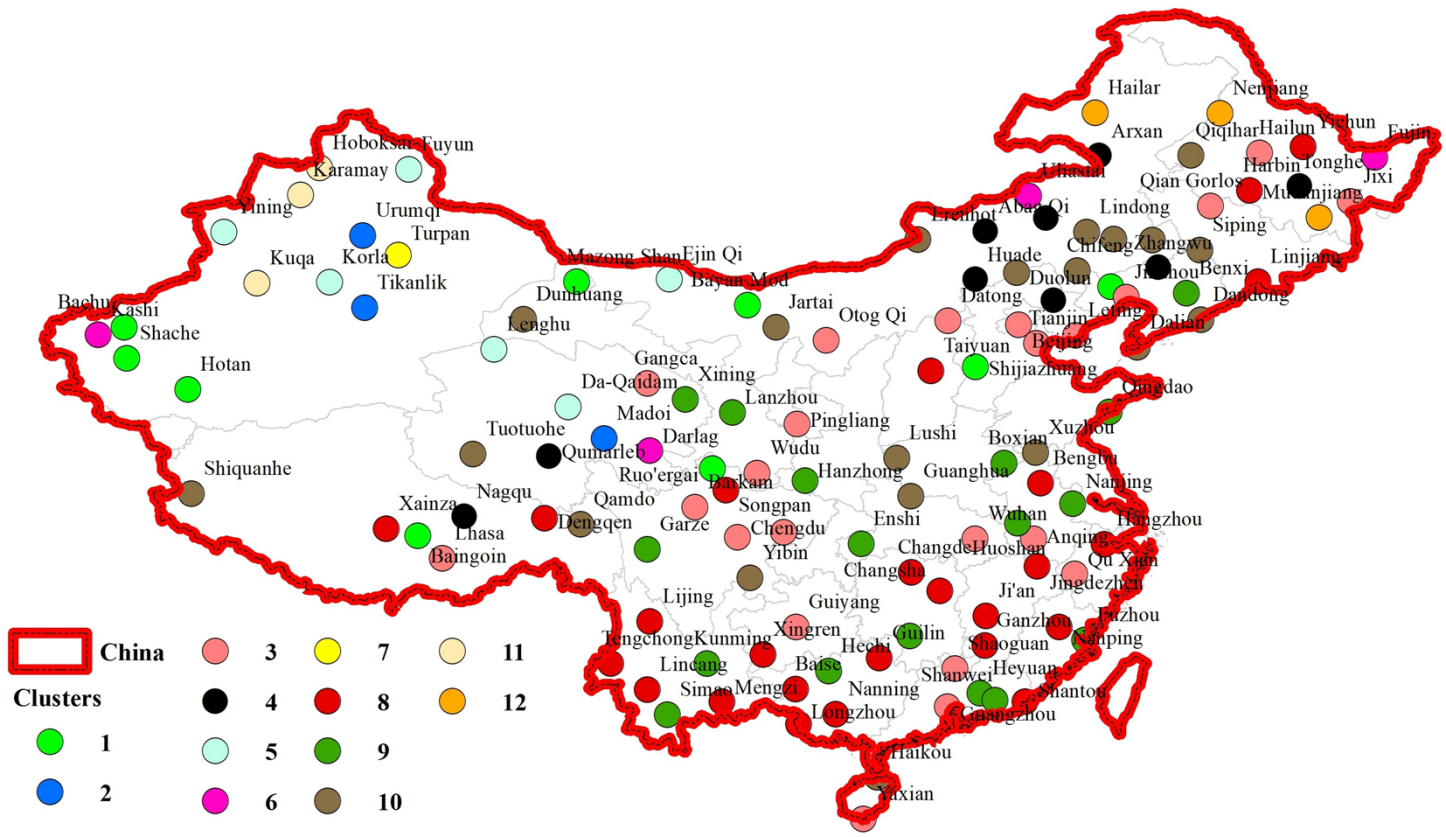
Clustering results through the suggested hybrid model based on MODWT-GNG in terms of the SC criterion values.

Figure 6 illustrates the clustering outcomes derived from a hybrid analytical model that integrates the Maximal Overlap Discrete Wavelet Transform (MODWT) with a Growing Neural Gas (GNG) algorithm, evaluated based on the SC (Silhouette Coefficient) criterion. In this approach, monthly precipitation data from synoptic stations across China were first decomposed into multiple time-scale sub-series using MODWT. Shannon entropy was then computed for each sub-series, reflecting the temporal complexity of precipitation at each location. The resulting entropy features served as inputs to the clustering process. Each colored dot on the map represents a synoptic station, with color indicating cluster membership. A total of 12 distinct clusters were identified, each corresponding to a unique precipitation variability profile based on both temporal scale and complexity. The spatial arrangement of clusters reveals meaningful regional patterns. For instance, clusters concentrated in southern and southeastern China (such as clusters 1 and 2) may represent stations with relatively stable and humid precipitation regimes. In contrast, clusters located in northern and northwestern regions (e.g., clusters 3, 4, and 7) likely correspond to areas experiencing higher variability and more complex precipitation dynamics, possibly due to the influence of diverse climatic systems, including continental and monsoonal effects. Moreover, several clusters (e.g., 6, 10, and 12) are distributed across transitional zones, suggesting intermediate levels of variability or mixed climatic influences. These areas may reflect regions with significant seasonal contrast or where abrupt shifts in precipitation regimes occur.

In an effort to better understand the identified clusters, we examined some of the major hydroclimatic characteristics of the stations in each of the 12 clusters. Clusters 1, 2, and 4 represent arid type regions with mean annual precipitation less than 300 mm, large inter-annual variability, and very short rainy seasons. Clusters 3 and 5 have patterns of moderate rainfall that typically begin in the fall, as well as high inter-annual variability of precipitation. Clusters 6 through 8 represent semi-humid/humid areas with annual total precipitation between 500 and 800 mm, and longer rainy seasons. Clusters 9 and 10 consist of stations from relatively high rainfall places where monthly and seasonal patterns are more consistent and repetitive heavy rainfall events occur. Clusters 11 and 12 had fewer stations, but appear to have a few distinguished rainfall regimes, possibly stronger teleconnection influences, or more extreme events. Overall, this classification provided a better understanding of the climatic environment in each cluster and informed future studies.

To improve the physical interpretation of clustering results, we linked the physical representation of the 12 identified clusters with the Köppen–Geiger climate classification zones using the updated global dataset from Beck et al.^40^. Each synoptic station was given a corresponding Köppen class and the dominant climate types were summarized.

Although we found a good match between specific clusters and predominant climatic regimes over China, it was observable that there are some clusters that had a distinct climate regime. For instance, clusters 1 and 2, which were primarily located in southeastern China, were primarily matched with humid subtropical (Cfa/Cwa) climate zones. Clusters 1 and 2 were primarily humid subtropical due to high levels of precipitation (high monthly mean) and low values for entropy. Clusters 3, 4, and 7, which are clustered across the northern and northwestern portions of China, cluster according to cold semi-arid (BSk) and arid (BWk) zones and therefore exhibited the largest transannual variability and highest entropy values.

Some clusters had some overlap transitional zones such as the 6 and 10 which crosses into temperate monsoonal (Dwa) or dry continental zones^40^. It is clear that the spatial clustering structure identified using entropy derived MODWT features validated against established climatic boundaries is indicative of the physical soundness of the framework utilized for clustering.

### Correlation between rainfall and teleconnection patterns

**Fig. 7:**
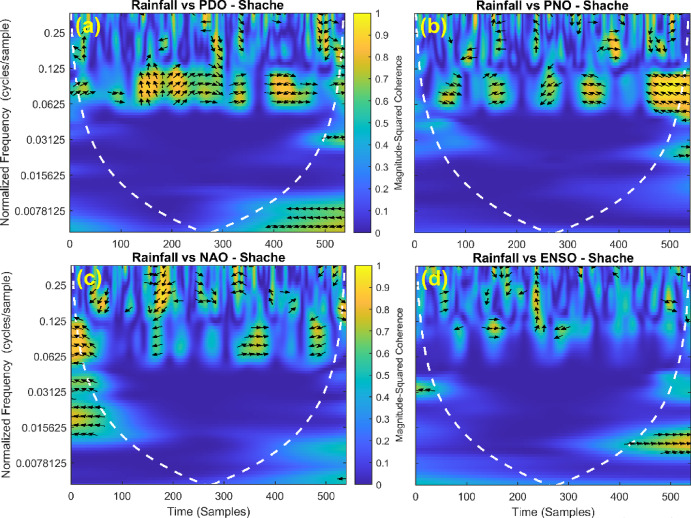
Time-frequency wavelet coherence between Shache station (cluster 1) rainfall and major climate indices (PDO, PNO, NAO, ENSO).

**Fig. 8:**
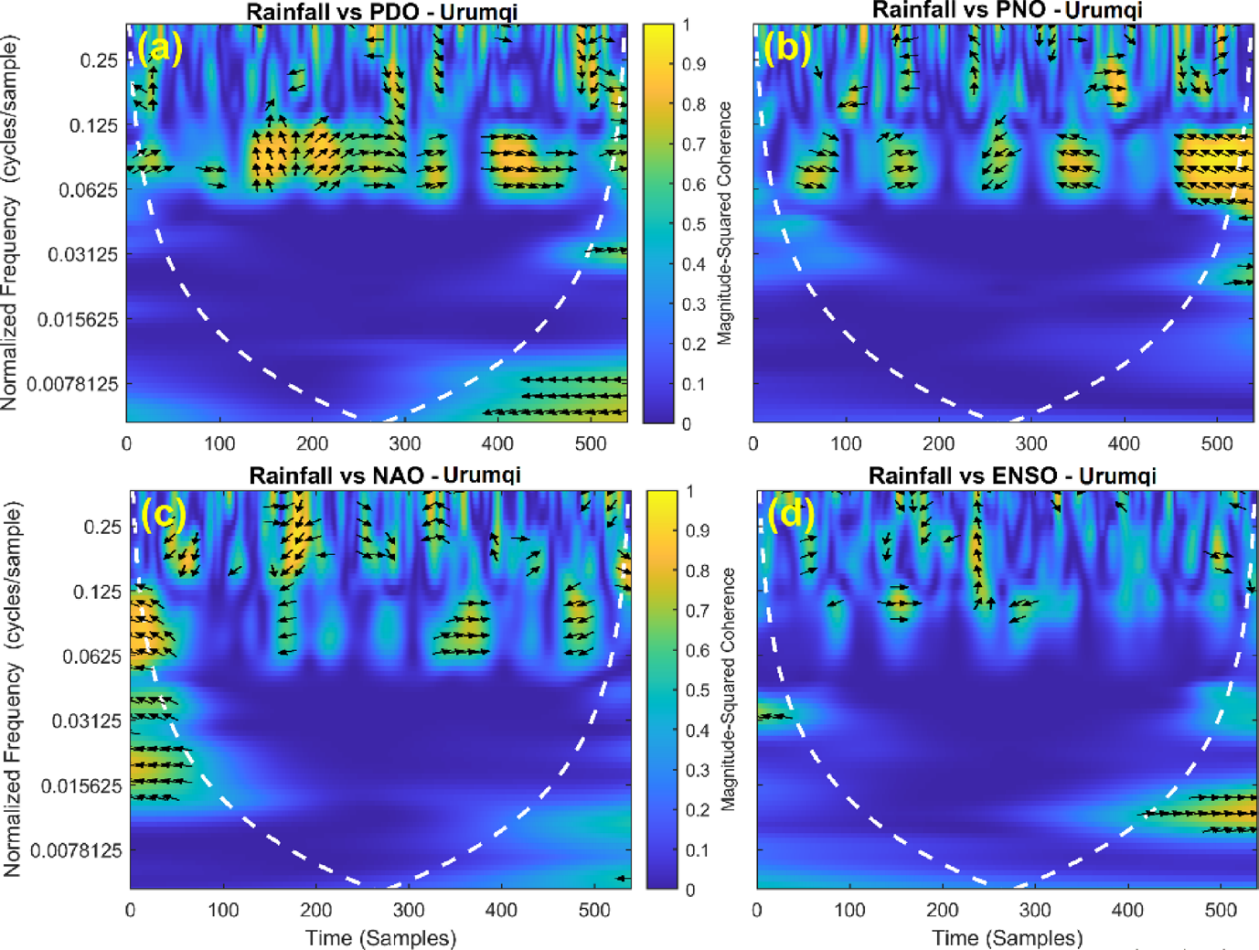
Time-frequency wavelet coherence between Urumqi station (cluster 2) rainfall and major climate indices (PDO, PNO, NAO, ENSO).

**Fig. 9:**
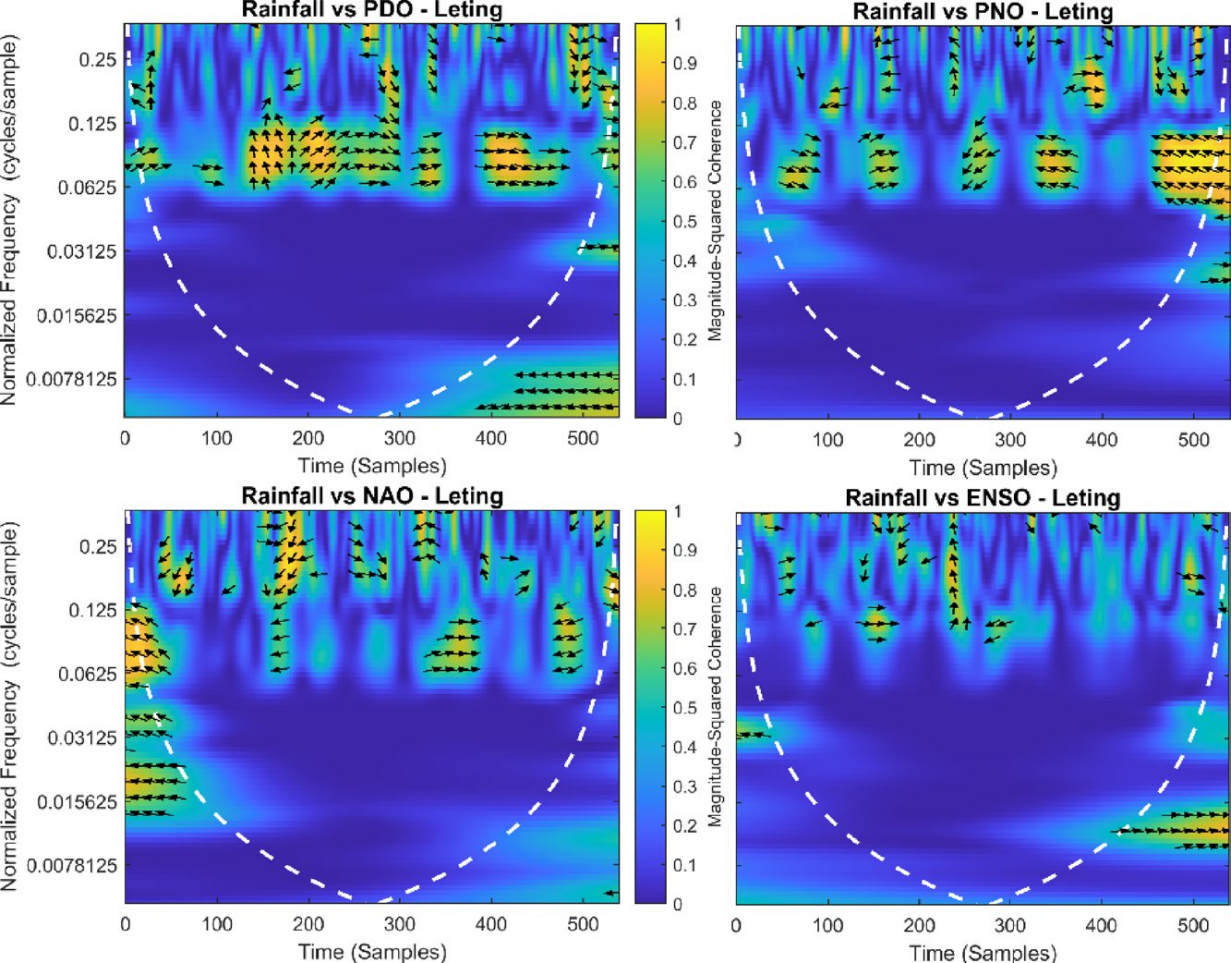
Time-frequency wavelet coherence between Leting station (cluster 3) rainfall and major climate indices (PDO, PNO, NAO, ENSO).

**Fig. 10:**
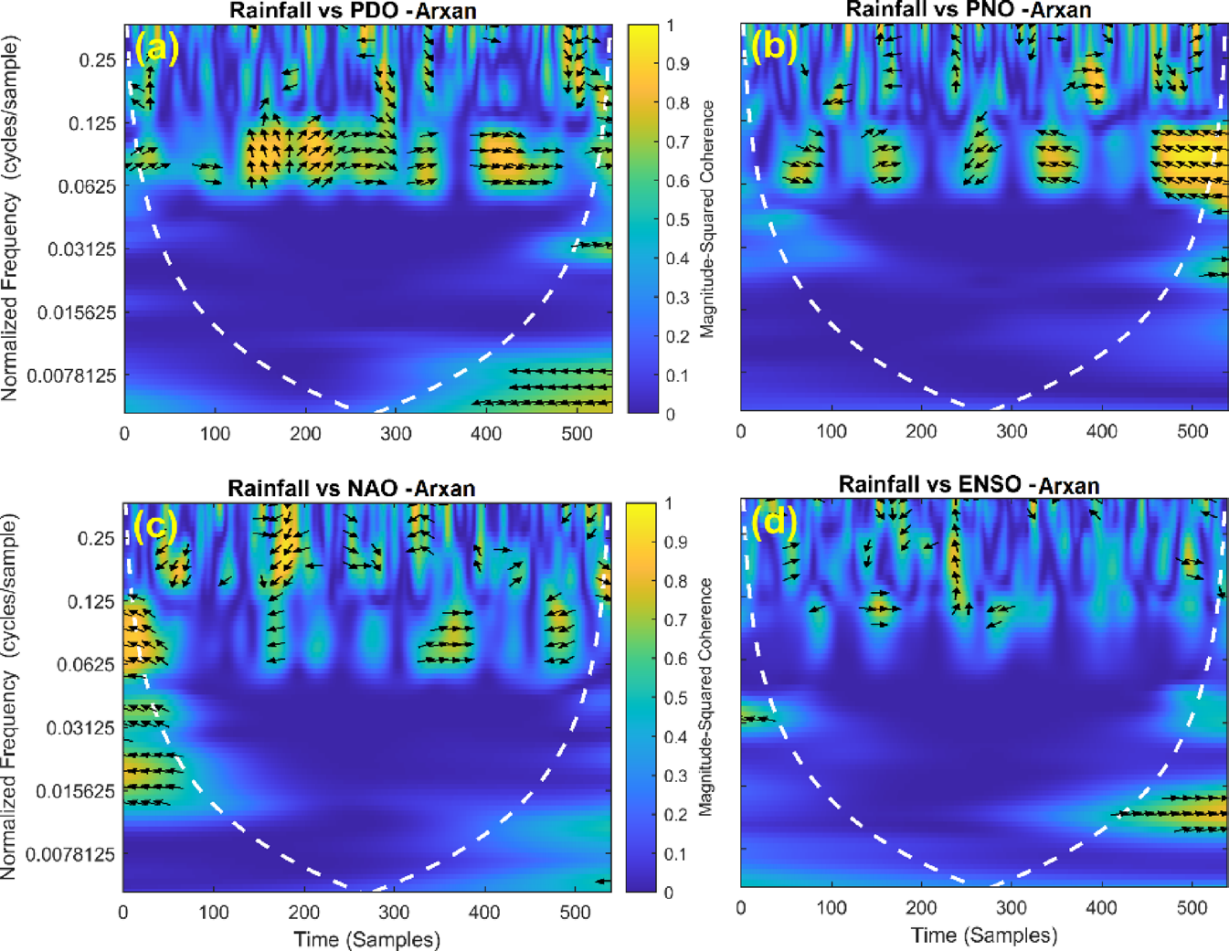
Time-frequency wavelet coherence between Arxan station (cluster 4) rainfall and major climate indices (PDO, PNO, NAO, ENSO).

**Fig. 11:**
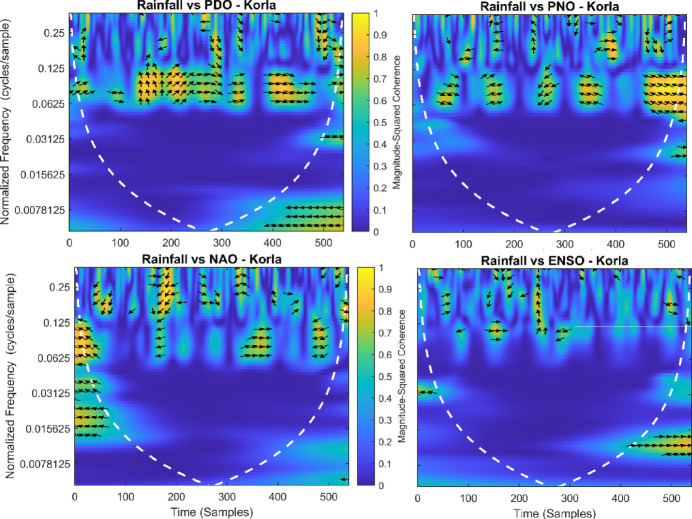
Time-frequency wavelet coherence between Korla station (cluster 5) rainfall and major climate indices (PDO, PNO, NAO, ENSO).

**Fig. 12:**
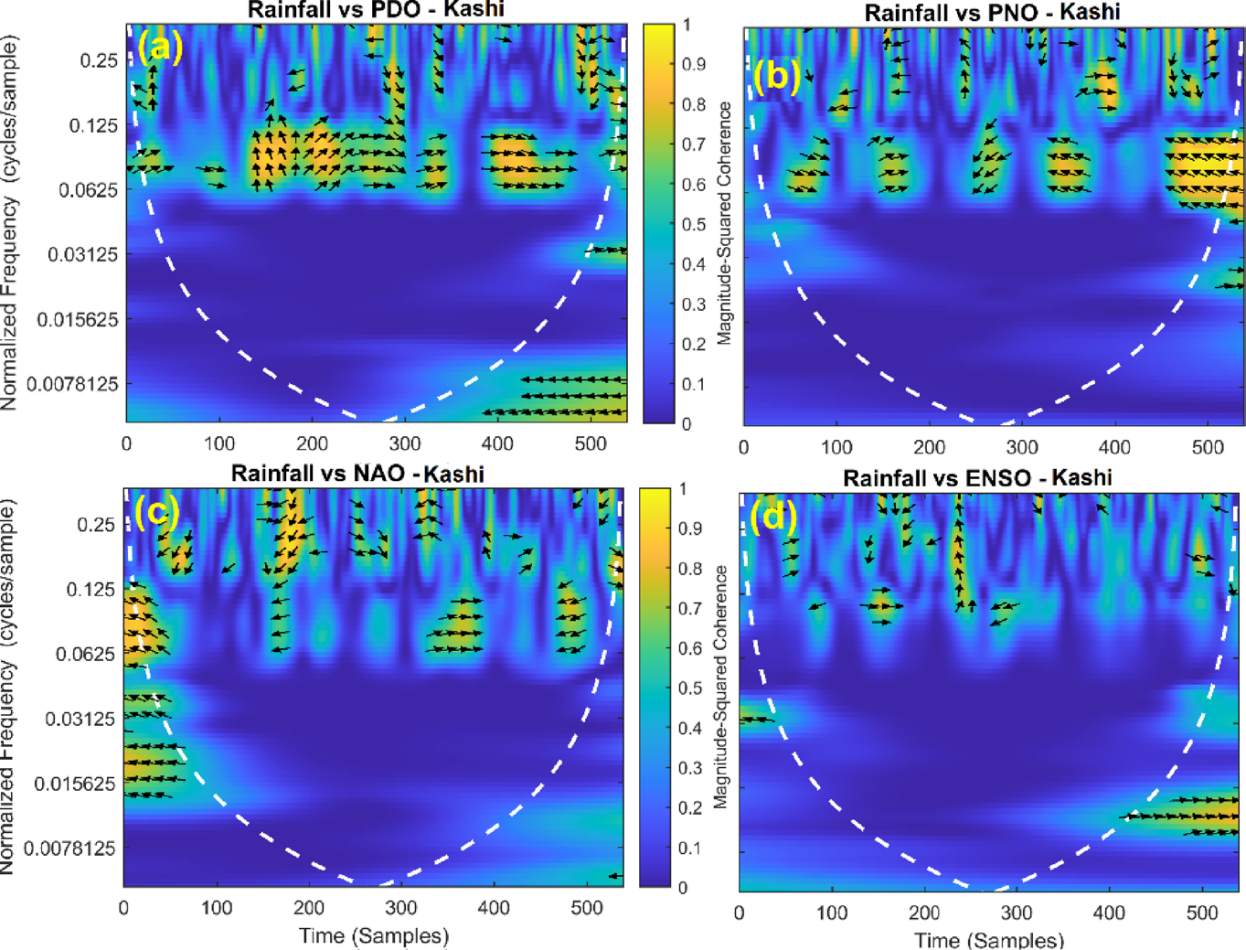
Time-frequency wavelet coherence between Kashi station (cluster 6) rainfall and major climate indices (PDO, PNO, NAO, ENSO).

**Fig. 13:**
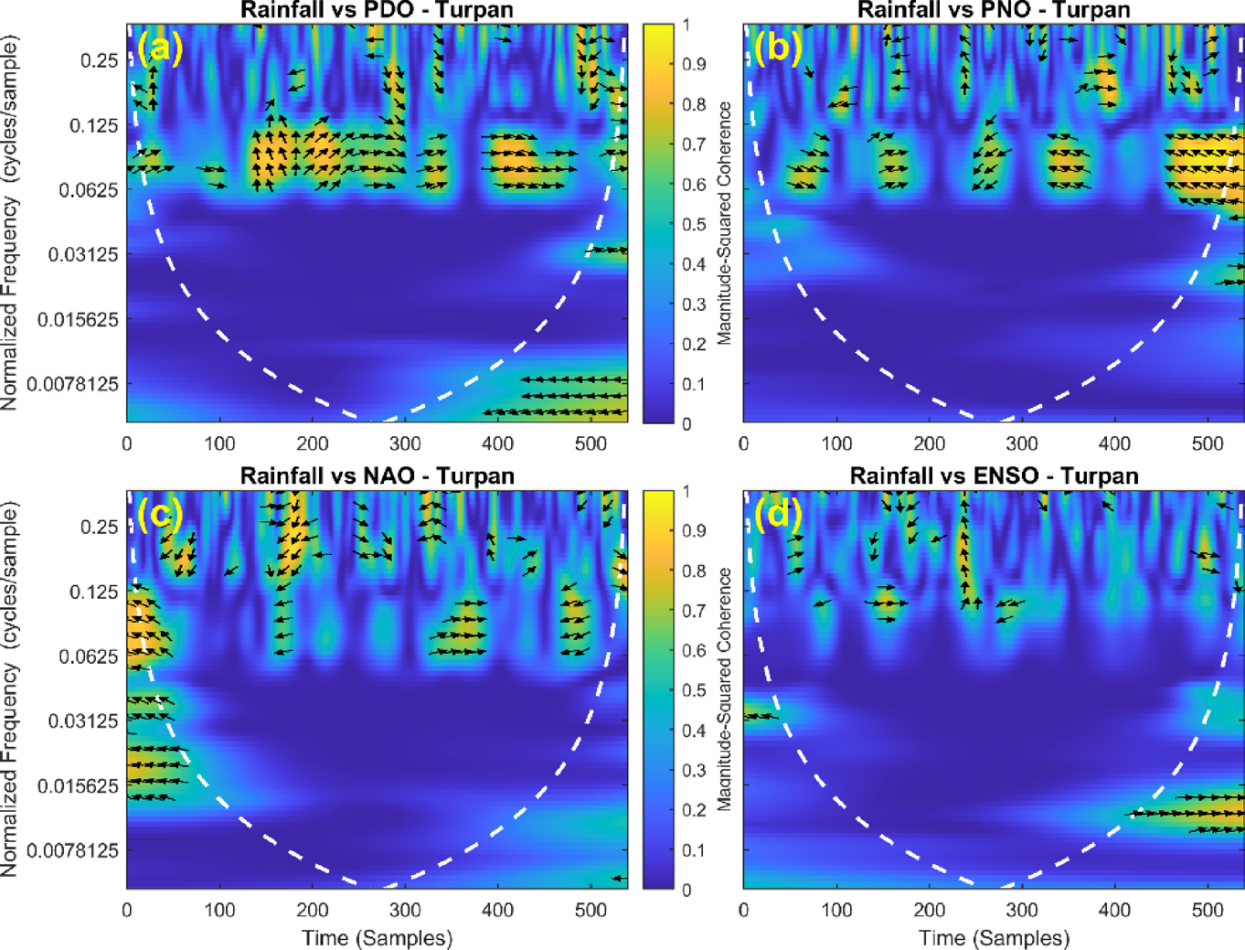
Time-frequency wavelet coherence between Turpan station (cluster 7) rainfall and major climate indices (PDO, PNO, NAO, ENSO).

**Fig. 14:**
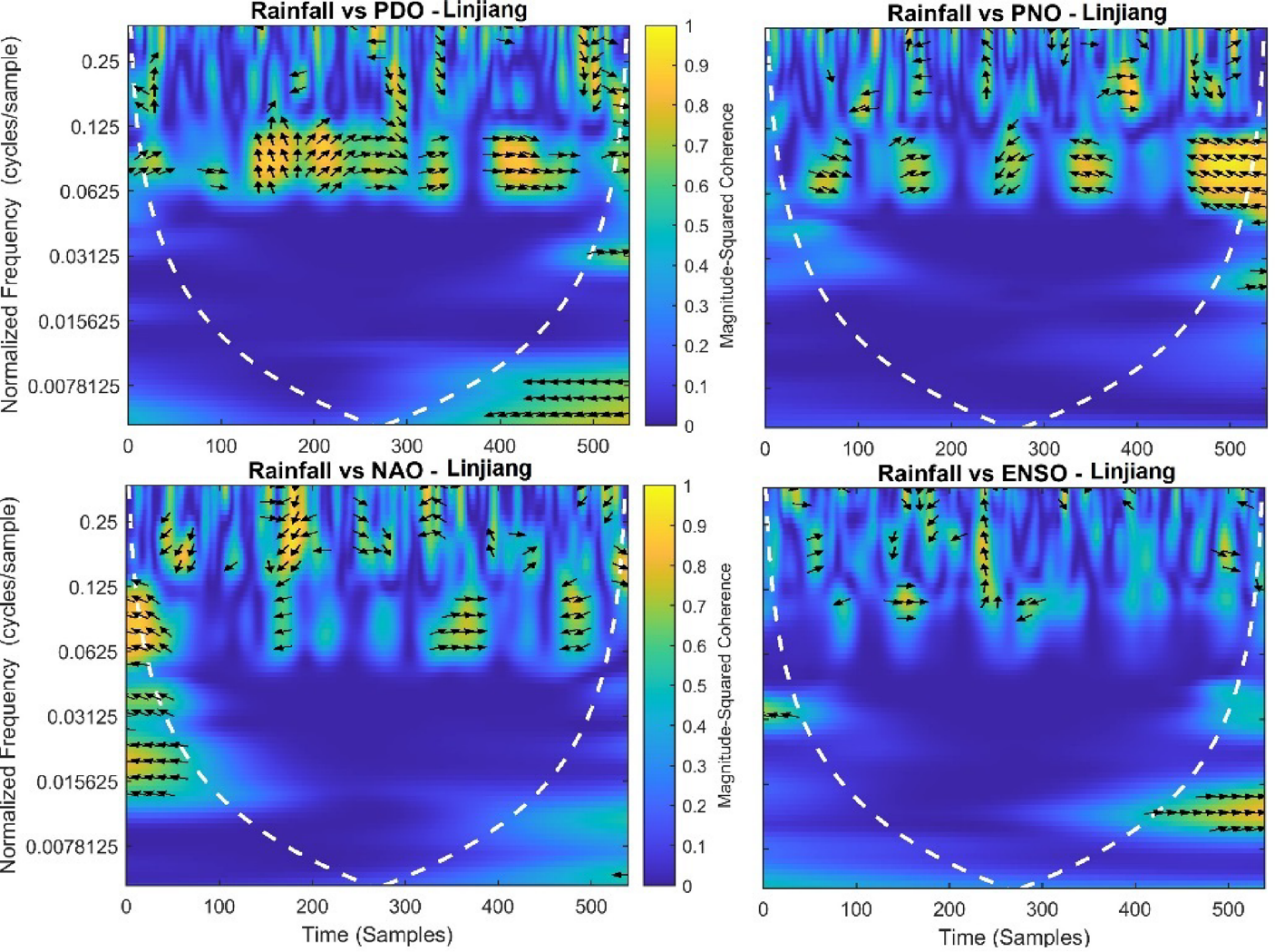
Time-frequency wavelet coherence between Linjiang station (cluster 8) rainfall and major climate indices (PDO, PNO, NAO, ENSO).

**Fig. 15:**
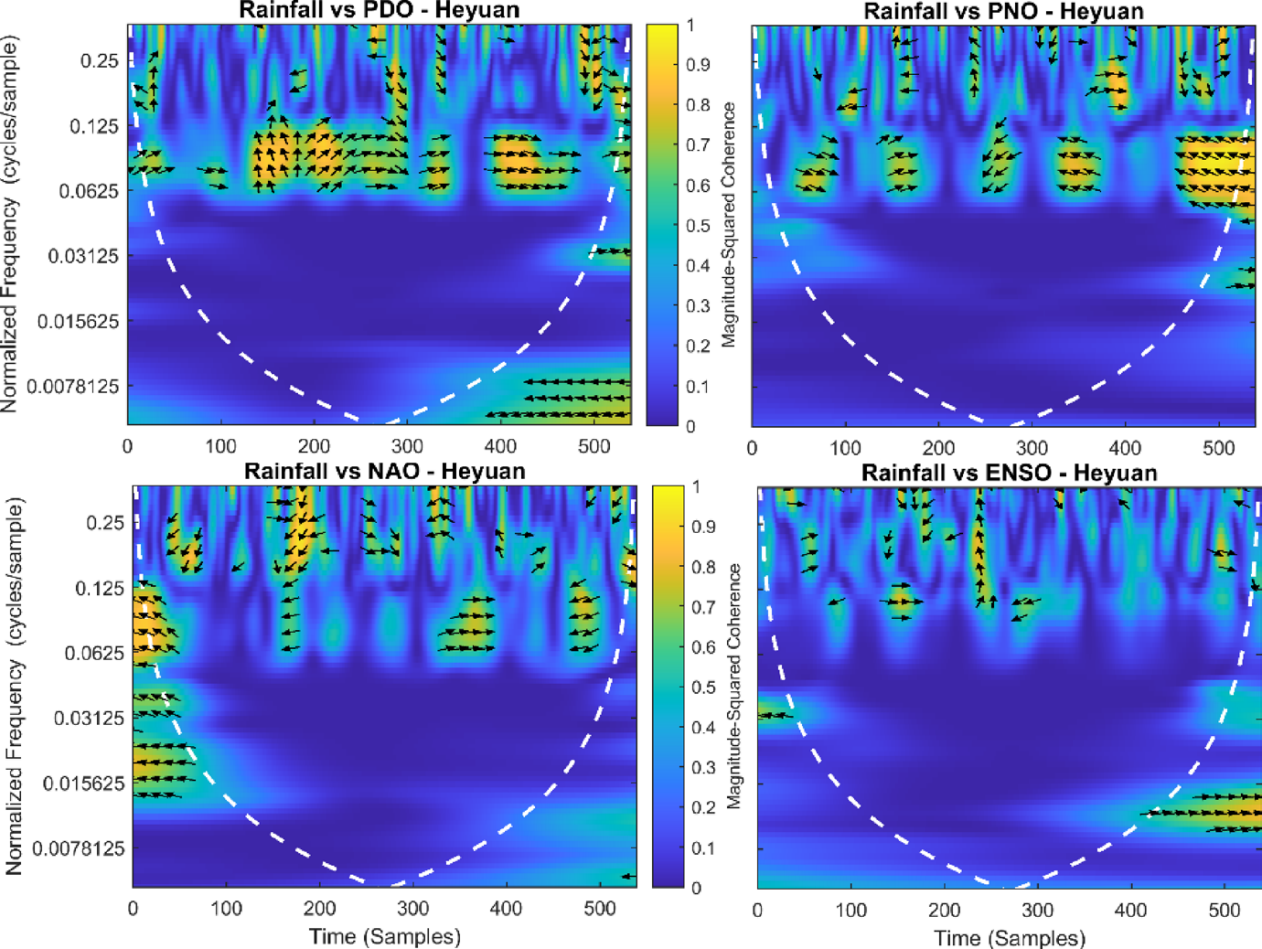
Time-frequency wavelet coherence between Heyuan station (cluster 9) rainfall and major climate indices (PDO, PNO, NAO, ENSO).

**Fig. 16:**
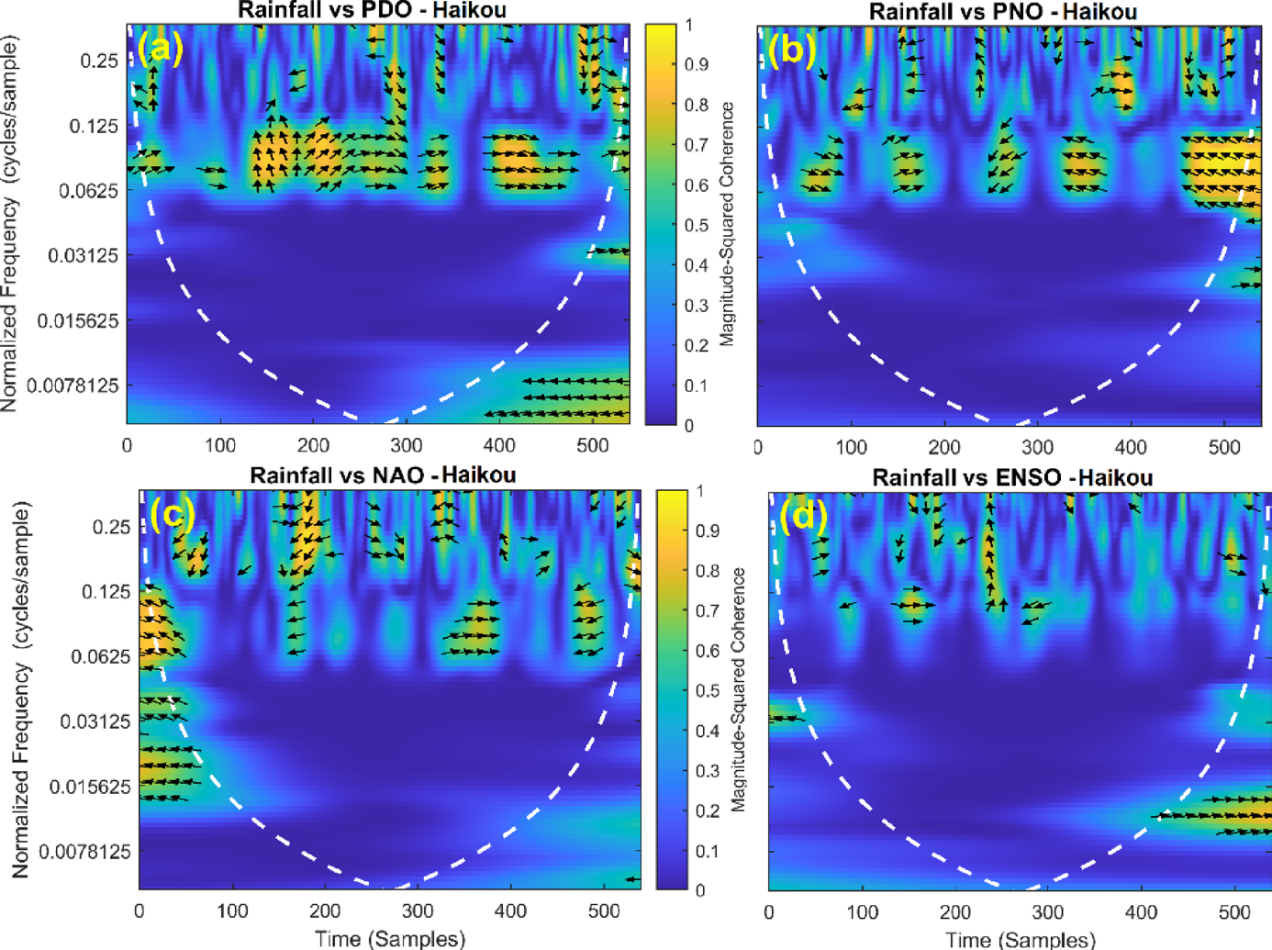
Time-frequency wavelet coherence between Haikou station (cluster 10) rainfall and major climate indices (PDO, PNO, NAO, ENSO).

**Fig. 17:**
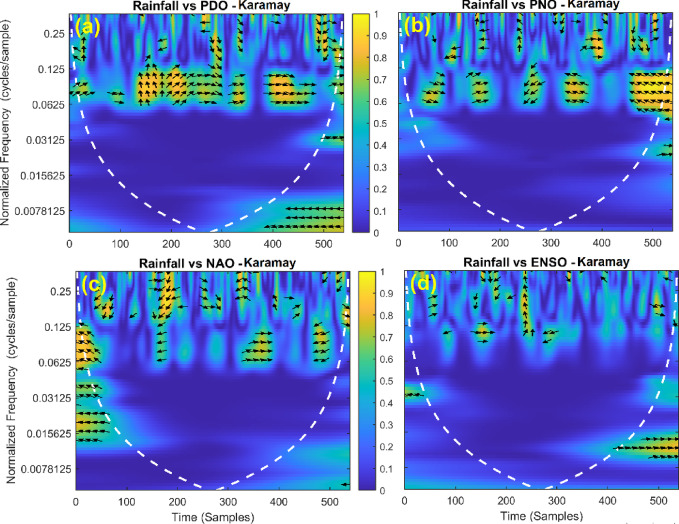
Time-frequency wavelet coherence between Karamay station (cluster 11) rainfall and major climate indices (PDO, PNO, NAO, ENSO).

**Fig. 18:**
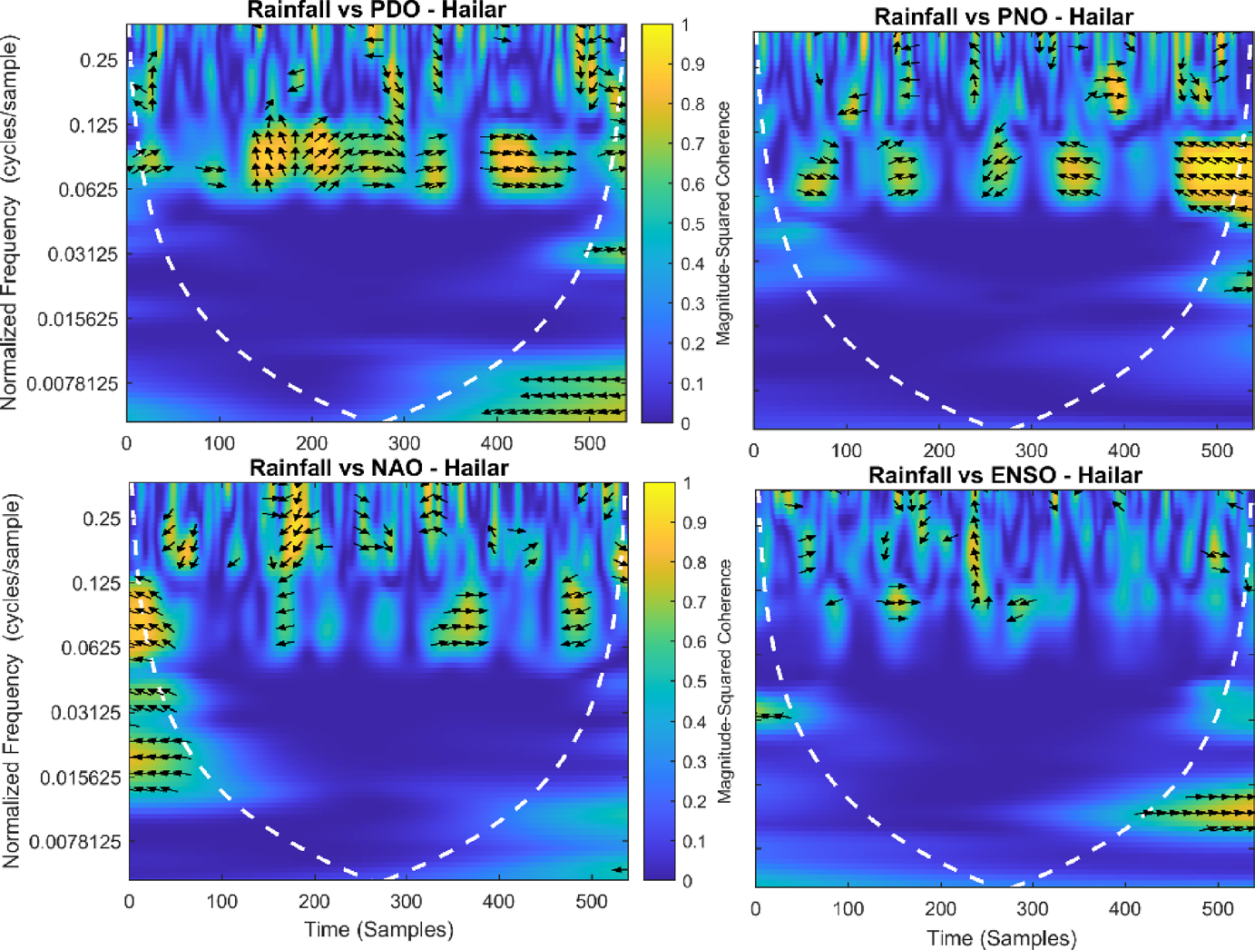
Time-frequency wavelet coherence between Hailar station (cluster 12) rainfall and major climate indices (PDO, PNO, NAO, ENSO).

This section provides a time-frequency wavelet coherence analysis for monthly rainfall in regions of China, as well as four leading large-scale climate oscillation indices - the Pacific Decadal Oscillation (PDO), Pacific/North American Oscillation (PNO), North Atlantic Oscillation (NAO), and El Niño–Southern Oscillation (ENSO) - and their relationships (Figs. 7, 8, 9, 10, 11, 12, 13, 14, 15, 16, 17 and 18). The regions selected for this study consist of a diversity of climatic and environmental characteristics from eastern monsoon regions to dry inland basins (e.g., Heyuan, Korla, Leting, Lhasa, Lijiang, Lingling, Nanjing, Shache, and Turpan). Overall, the formally specified climate oscillation indices (i.e., PDO and NAO) display the most persistent and coherent relationship with regional rainfall (e.g., they are in phase) through a range of mid-frequency bands (e.g., 0.0625–0.125 cycles/sample at most locations). As is expected, this substantial relationship between rainfall and coherent associations is likely related to the modulation of sea level pressure which results in changes in sea surface temperature and the atmospheric circulation patterns affecting the East Asian monsoon and mid-latitude westerlies or other teleconnections. On the other hand, the PNO and ENSO indices produce less coherent and episodical relationships with monthly rainfall, and significant indices within the overall context of time intervals or frequency bands that lead to the presence of coherence. When is relevant, the ENSO index tends to lead the monthly rainfall variability with a time lag consistent with teleconnection processes, such as the delayed influence of ENSO on the Walker circulation and changes in subtropical jet streams. Each teleconnection - the PDO, PNO, NAO, and ENSO, interact with each other, influencing rainfall over space (i.e., regions in the study, and time). Rainfall in eastern monsoon regions can lead to coherent future influences for rainfall regions further inland and a few months into the future. While there are regional variations, a common theme is evident: the timing and strength of climate-rainfall coherence varies by climate mode and region. While highland and inland areas such as Lhasa and Turpan show more intermittent yet meaningful coherence, eastern areas such as Nanjing and Leting show a relatively wider and more stable teleconnection. In general, the findings emphasize that persistent and transient climate drivers must be recognized together in rainfall variability across China. The results have applied importance in facilitating seasonal rainfall forecasting, drought monitoring and planning of long-term water resources.

Table 2 is a summary of the prevalent teleconnection patterns with rainfall variability in the 12 clusters. According to the findings in the table, the significance of large-scale climate oscillations like PDO and NAO dominate the coherence of monthly rainfall among various clusters in China. PDO, in particular, is observed in the majority of the stations such as Shache, Urumqi, Leting, Arxan, Korla, Kashi, Turpan, Heyuan, and Karamay, with moderate to strong and persistent coherence mainly in the mid-frequency band (0.0625–0.125 cycles/sample). At certain stations, including Shache, Leting and Kashi, displays of both strong and stable coherence with PDO and NAO at the same time show that these indices exert a consistent effect on local rainfall variations. In the meantime, the PNO index demonstrates strong to moderate and widespread coherence in such stations as Linjiang and Haikou, especially in eastern areas where the coherence is more resilient with time. There are stations that exhibit a low level of coherence with intermittence and episodic characteristics, i.e. short- term and regional influences of the oscillation indices, e.g., Turpan and Haikou. All in all, the coherence is largely confined in the middle frequency, with the effects of PDO and NAO being more coherent and regionally meaningful, but the effects of PNO being more localized to eastern and coastal regions.

**Table 2:** Dominant teleconnection indices influencing rainfall variability and their coherence characteristics across the 12 clusters in China.

Cluster/Station	Dominant teleconnection(s)	Strength of coherence	Frequency band (cycles/sample)	Temporal characteristic
Cluster 1 – Shache	PDO, NAO	Strong & persistent	0.0625–0.125	In-phase, mid-frequency
Cluster 2 – Urumqi	PDO	Moderate–strong	0.0625–0.125	Stable, mid-frequency
Cluster 3 – Leting	PDO, NAO	Strong & stable	0.0625–0.125	Persistent in eastern region
Cluster 4 – Arxan	PDO	Moderate	0.0625–0.125	Episodic but recurrent
Cluster 5 – Korla	PDO	Moderate	0.0625–0.125	In-phase, mid-frequency
Cluster 6 – Kashi	PDO, NAO	Strong	0.0625–0.125	Stable mid-frequency
Cluster 7 – Turpan	PDO	Intermittent	0.0625–0.125	Episodic, meaningful
Cluster 8 – Linjiang	PNO, PDO	Strong	0.0625–0.125	Broad, stable coherence
Cluster 9 – Heyuan	PDO	Moderate	0.0625–0.125	In-phase, mid-frequency
Cluster 10 – Haikou	PDO, PNO	Weak–moderate	Episodic	Episodic, meaningful
Cluster 11 – Karamay	PDO	Moderate	0.0625–0.125	Regional persistence
Cluster 12 – Hailar	PDO, NAO	Moderate–strong	0.0625–0.125	Mid-frequency coherence

## Discussion

The results of this study emphasize the significance of incorporating temporal decomposition and entropy analysis prior to spatial clustering of precipitation data. By applying the Maximal Overlap Discrete Wavelet Transform (MODWT), key temporal dynamics across different frequency bands were successfully captured, revealing patterns often obscured in raw time series. The entropy values derived from these decomposed components reflected complex variability in regions such as northern and northeastern China, where diverse climatic influences prevail. Subsequently, processing these entropy features through the Growing Neural Gas (GNG) algorithm enabled spatial clustering that identified twelve distinct regions, each characterized by unique precipitation variability signatures. Notably, clusters in southern China exhibited more stable and consistent rainfall patterns, while northern regions demonstrated higher variability and complexity. Transitional zones displayed intermediate behavior, underscoring the sensitivity of the proposed approach to subtle climatic gradients. Compared to conventional clustering techniques, the combined MODWT-GNG framework provided improved spatial coherence and interpretability. The silhouette coefficient (SCi = 0.68) confirmed the robustness of this hybrid method in distinguishing homogeneous regions, outperforming clustering conducted on the original, unprocessed data. These findings indicate that the integration of multiscale temporal decomposition with neural network-based clustering constitutes a powerful tool for hydrological pattern recognition and regional climate analysis. This approach aligns with and extends findings from prior studies. For example, Hsu and Li (2010) utilized a combination of wavelet transform and Self-Organizing Maps (SOM) to capture scale-dependent and temporal dynamics in precipitation data, successfully delineating hydrologically homogeneous regions^37^. Similarly, Zhang et al.^41^ employed entropy-based indices to analyze spatiotemporal precipitation patterns within the Huai River Basin in China, identifying areas with analogous rainfall variability. Furthermore, Sun and Niu^42^ applied wavelet-based multiscale entropy to delineate soil moisture dynamics, demonstrating the method’s capability to detect homogenous clusters based on complex temporal behavior. In addition, Zhang et al.^43^ used optimized sample entropy to assess spatial complexity in precipitation across Heilongjiang Province, revealing significant regional heterogeneity.

Areas within clusters that had high wavelet entropy and high interannual variability in precipitation most likely represent unstable and complicated climates driven by external factors, typically large scale climatic drivers; namely El Niño Southern Oscillation (ENSO) or Pacific Decadal Oscillation (PDO). In these scenarios, the predictability of rainfall is decreased, and the likelihood of extreme events increases, with substantial variability in water availability as a result. As such, these regions should favor adaptive and flexible policy tools. Adaptive strategies such as dynamic water allocation (based on seasonal forecasts), risk-based drought and flood management, flexible management of surface and ground waters, or even diversifying water supply options (via recycling or desalination) can help improve resilience in areas susceptible to climate variability. Furthermore, if possible, an immediate real time assessment, monitoring system, or early warning system would also be prudent in these areas of intense risk. Conversely, clusters with lower wavelet entropy and generally more stable precipitation regimes may exhibit characteristics such as seasonal regularity in precipitation (or drought), stable annual water supply, and a higher reliance on regional climate cycles. In these areas, long-term and enduring infrastructure types of planning could be more effective approaches to sustainable water resource management. For example, policies related to the construction of large reservoirs related to flow management, the construction of high-efficiency irrigation networks, developed managed aquifer recharge systems, and effective waters use efficiency and conservation programs (agriculture and urban) can make water use sustainable and reliable across all sectors. For demand-side management, particularly in the case of rainfed agriculture, management can also be more practical and effective given less variability in the climate. Overall, this distinction between clusters can be used as a tool for spatially differentiated water policy development, and allow each cluster a management framework that reap good line with the areas hydroclimatic characteristics. This also makes for more efficient decisions within the allocation of all water resources, while enhancing the resilience of water systems to climate variability and uncertainty over time.

Collectively, these studies corroborate the efficacy of combining multiscale decomposition techniques with advanced clustering algorithms for capturing intricate hydrological and climatic patterns. The present study’s use of MODWT in conjunction with the GNG algorithm advances this methodology by enhancing spatial classification accuracy and interpretability of precipitation variability across diverse climatic zones.

## Conclusion

This study developed and validated a hybrid approach for precipitation regionalization by integrating MODWT and the Growing Neural Gas algorithm. The method effectively extracted temporal variability patterns from long-term precipitation series and utilized them to identify twelve homogeneous precipitation zones across China. The application of Shannon entropy to MODWT sub-series allowed for the incorporation of multiscale dynamics into the clustering process, leading to improved spatial classification results. The enhanced performance of the proposed model—reflected in the optimal silhouette score—demonstrates its suitability for analyzing complex hydrological datasets. This methodology can assist policymakers and water managers in designing adaptive strategies for flood control, drought preparedness, and sustainable water resource allocation under changing climatic conditions.

However, several limitations should be acknowledged. First, the analysis relied solely on precipitation data, while other hydroclimatic variables such as temperature, evapotranspiration, and land surface characteristics were not incorporated, which may influence regional homogeneity. Second, although the MODWT-GNG framework effectively captures multiscale variability, the choice of wavelet basis and entropy measure can affect the sensitivity of the clustering results. Additionally, the spatial generalization of the findings may be constrained by the resolution and coverage of the observational dataset. Future studies could expand the approach by incorporating multi-variable datasets and testing its applicability across different geographic regions and climate regimes.

## Data Availability

The precipitation data used in this study was obtained from the Meteostat site, (https://meteostat.net/en/), which gathers and standardizes climate observations from multiple national and international meteorological agencies. Monthly cumulative precipitation data, from January 1980 through December 2024, were downloaded for 123 synoptic weather stations in China, resulting in 540 months of total data for each station. The location of the synoptic weather stations was chosen to provide the best spatial representation of the different climatic zones-geographical areas, (i.e. arid desert in the northwest to humid subtropical zones in the southeast). To preserve data quality, only records with uninterrupted observations for the full study period were used and non-parametric Run tests (*p* < 0.05) were used to analyze temporal consistency and homogeneity of all-time series to ensure they were suitable for long-term climate records. The dataset’s structure ensures broad spatial and temporal coverage, and presents the statistical distribution of annual precipitation values, which highlight substantial climatic heterogeneity: less than 50 mm annually in the arid northwest, more than 2000 mm in the southeastern coastal regions, and a national mean of approximately 670 mm over the study period-slightly higher than the official estimate of 645 mm reported by the China Meteorological Administration (CMA).
