# Correction: A hybrid approach for regionalization of precipitation based on maximal discrete wavelet transform and growing neural gas network clustering

**DOI:** 10.1038/s41598-025-33156-7

**Published:** 2026-01-16

**Authors:** Xu Tao, Ma Ben, He Cao Yin Xuan, Ali Arshaghi

**Affiliations:** 1 College of Mechanical and Electrical Engineering, Wuyi University, Wuyishan, 354300 China; 2 The Key Laboratory for Agricultural Machinery Intelligent Control and Manufacturing of Fujian Education Institutions, Wuyi University, Wuyishan, 354300 China; 3 Department of Electrical Engineering, CT.C., Islamic Azad University, Tehran, Iran

Correction to: *Scientific Reports* 10.1038/s41598-025-24400-1, published online 18 November 2025

The original version of this Article contained an error in Figures 1, 5 and 6 where the figures were inadvertently generated using data prior to the preprocessing stage. As a result, these figures did not accurately represent the methodology workflow, the post-processing model inputs, or the final precipitation regionalization results as described in the manuscript. The original Figures 1, 5 and 6 and accompanying legends appear below.

The original Article has been corrected.

**Fig. 1:**
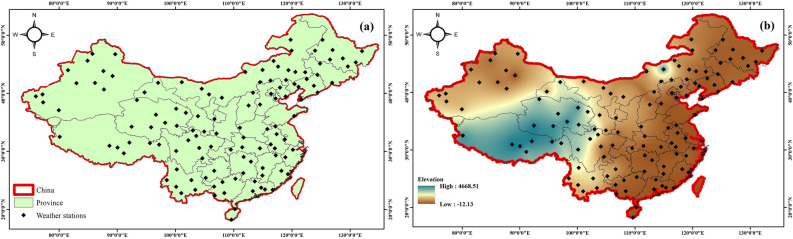
(**a**) Map of China’s location of synoptic stations. (**b**) Map of China’s Digital elevation model.

**Fig. 5:**
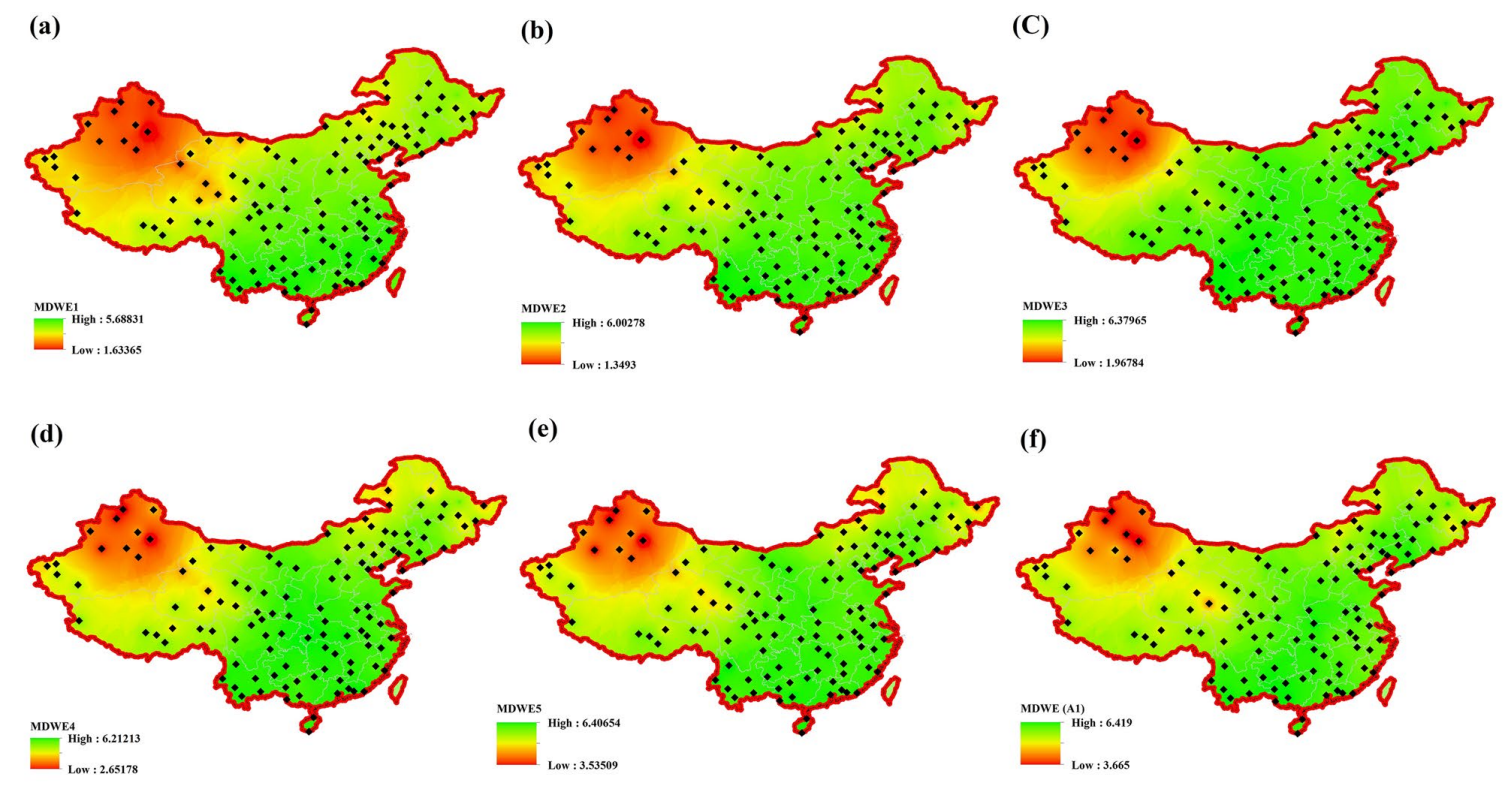
Spatial distribution of monthly wavelet entropy (MDWE) across China based on MODWT-decomposed precipitation sub-series at different time scales.

**Fig. 6:**
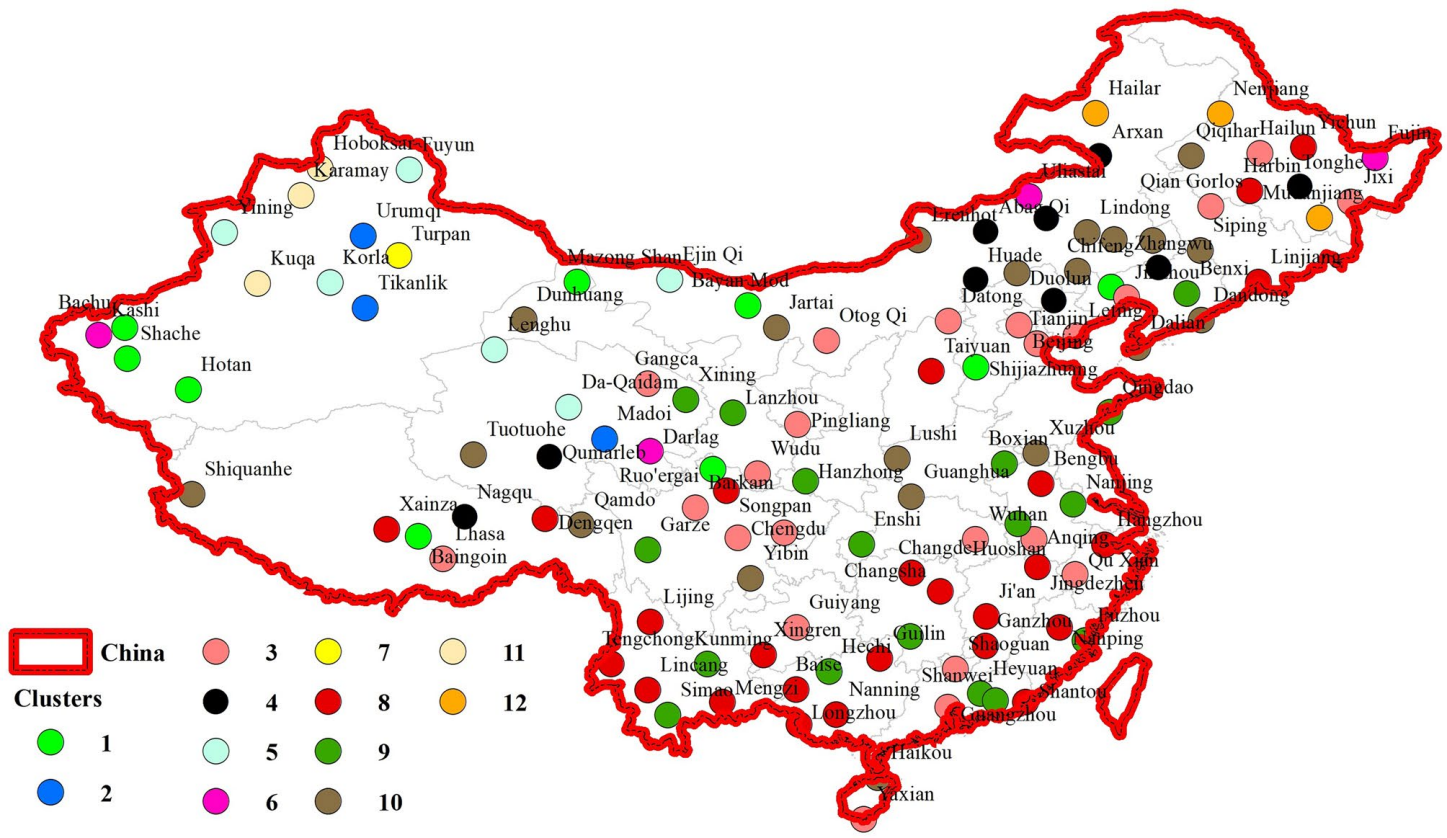
Clustering results through the suggested hybrid model based on MODWT-GNG in terms of the SC criterion values.

